# Membrane nanotubes transform into double-membrane sheets at condensate droplets

**DOI:** 10.1073/pnas.2321579121

**Published:** 2024-06-20

**Authors:** Ziliang Zhao, Vahid Satarifard, Reinhard Lipowsky, Rumiana Dimova

**Affiliations:** ^a^Max Planck Institute of Colloids and Interfaces, Potsdam 14476, Germany; ^b^Leibniz Institute of Photonic Technology e.V., Jena 07745, Germany; ^c^Institute of Applied Optics and Biophysics, Friedrich-Schiller-University Jena, Jena 07743, Germany; ^d^Yale Institute for Network Science, Yale University, New Haven, CT 06520

**Keywords:** tube-to-sheet transformation, double-membrane sheet, giant unilamellar vesicles (GUV), stimulated emission depletion (STED), condensate interface

## Abstract

Cellular membranes exhibit a multitude of highly curved morphologies such as buds, nanotubes, cisterna-like sheets defining the outlines of organelles. Here, we mimic cell compartmentation using an aqueous two-phase system of dextran and poly(ethylene glycol) encapsulated in giant vesicles. Upon osmotic deflation, the vesicle membrane forms nanotubes, which undergo surprising morphological transformations at the liquid–liquid interfaces inside the vesicles. At these interfaces, the nanotubes transform into cisterna-like double-membrane sheets (DMS) connected to the mother vesicle via short membrane necks. Using super-resolution (stimulated emission depletion) microscopy and theoretical considerations, we construct a morphology diagram predicting the tube-to-sheet transformation, which is driven by a decrease in the free energy. Nanotube knots can prohibit the tube-to-sheet transformation by blocking water influx into the tubes. Because both nanotubes and DMSs are frequently formed by cellular membranes, understanding the formation and transformation between these membrane morphologies provides insight into the origin and evolution of cellular organelles.

## Significance

The endoplasmic reticulum (ER) is a vital cellular structure comprising interconnected tubules and double-membrane sheets (cisternae). While various biological functions of the ER are directly related to these highly curved structures, the mechanisms behind their formation and transformation remain poorly understood. Our research brings biophysical insights into these processes by highlighting the potential role of biomolecular condensates and wetting phenomena. Using cell mimetic systems, we provide evidence that wetting of droplets on the membranes plays a key role in maintaining and interconversion of the two types of membrane structures, surprisingly even in the absence of membrane scaffolding proteins. These findings offer insights into the biophysics of cellular organization and function.

To perform their functions, cellular membranes adopt a variety of highly curved morphologies. Nanotubes and disk-like cisterna (or double-membrane sheets, DMS) are pivotal examples of the palette of membrane shape elements. They are characterized by a high area-to-volume ratio, enhancing membrane-dependent processes and offering an efficient way to store membrane area for future usage such as vesicular transport between the endoplasmic reticulum (ER) cisternae and the Golgi apparatus (1, 2). The ER is among the most architecturally striking eukaryotic organelles, representing a single interconnected membrane system hosting the nuclear envelope, sheet-like cisternae, and a network of tubules connected by three-way junctions (3). The nuclear envelope consists of two continuous membrane sheets (inner and outer) that are interconnected by nuclear pores. The globular shape of the nuclear envelope is stabilized by interactions between its inner nuclear membrane proteins and the underlying chromatin (4) or the nuclear lamina (5) in higher eukaryotes. Sheets and tubules have distinct characteristic curvature, which is high along the tubes and sheets periphery, shaped and stabilized by special proteins (3) as in the nuclear envelope. Curvature-stabilizing proteins include reticulons and DP1/Yop1p (4, 6), while the separation of two sheets is believed to be maintained by “bridging” proteins that act as luminal spacers (3, 7). Although super-resolution microscopy has revealed the nanoscale dimensions of ER tubules and sheets in living cells (8), the driving forces generating and transforming these structures remain elusive. Increasing evidence points to a close relationship between the assembly and morphology of condensates and the ER (9–12). Recent studies report that even the morphology of biomolecular condensates is regulated by ER sheet proliferation at the expense of tubules (13). Intracellular nanotubes connect distant parts of the cell for transport and signaling (14), while intercellular nanotubes are responsible for cell–cell communication, transferring, and releasing cellular content (15–18). Their length can exceed several fold the cell size (19) while their thickness is below conventional optical resolution. The diameters either of pulled (20) or moving tubes (21) in simplified mimetic cell models can be directly assessed in vitro using STED (stimulated emission depletion) microscopy. Recent studies have shown that viruses including coronaviruses (SARS-CoV) can reproduce via a replication membrane compartment formed from the ER membrane, either by invagination toward the ER lumen (employing DMSs) or extrusion originating from the ER (forming double-membrane vesicles) (22, 23). Similar processes utilizing ER membrane replication have facilitated the production of SARS-CoV-2 virus-like particles, lifting the constraint of conducting coronavirus research exclusively in biosafety level 3 laboratories (24, 25). The formation and scission mechanisms of these double-membrane structures during replication at the ER sites are still poorly understood (26).

In this study, we present a hypothesis for the mechanism of DMS formation. Using a cell-mimetic system with internal condensate-based compartmentation, we observe and capture at video frequency tube-to-sheet transformations at the condensate interface. Nanotubes produced by vesicle deflation transform into DMS reminiscent of ER cisternae-structures as characterized by both two- and three dimensional STED. Two possible pathways for this transformation are resolved. A morphology diagram based on wetting theory predicts correctly the tube-to-sheet transformation. Nanotube knots formed by tube entanglement can prohibit this transformation but can also coexist with preexisting membrane sheets. Our system of coexisting nanotubes and DMS resembles ER structures, albeit in the absence of membrane proteins. As nanotubes and membrane sheets are important functional cellular structures, exploring their transformation, structure, and coexistence is of vital importance for elucidating the origin and evolution of these highly curved morphologies during cellular events. Our research suggests the importance of wettability by biomolecular condensates in modulating certain cellular membrane functions.

## Results and Discussion

### DMS Formation and 3D Reconstruction Models.

As cell-mimetic membrane compartments, we explored giant unilamellar vesicles (GUVs) (27, 28) made of a ternary lipid mixture in the liquid-disordered phase (Ld) (*Methods*). The vesicles encapsulated dextran/PEG (poly(ethyleneglycol)) aqueous two-phase system (ATPS) in the one-phase region with a dextran-to-PEG weight ratio D/P = 1.57 (4.76% and 3.03% weight fractions). The GUVs were trapped and immobilized in a microfluidic device (21, 29) (see *Methods* and *SI Appendix*, Fig. S1 for more details). Deflation was performed using dextran/PEG solutions of D/P = 1 (3.54%, 3.54% weight fraction) containing increasing amounts of sucrose, thereby applying a 20% stepwise increase in exterior osmolarity.

**Fig. 1.**
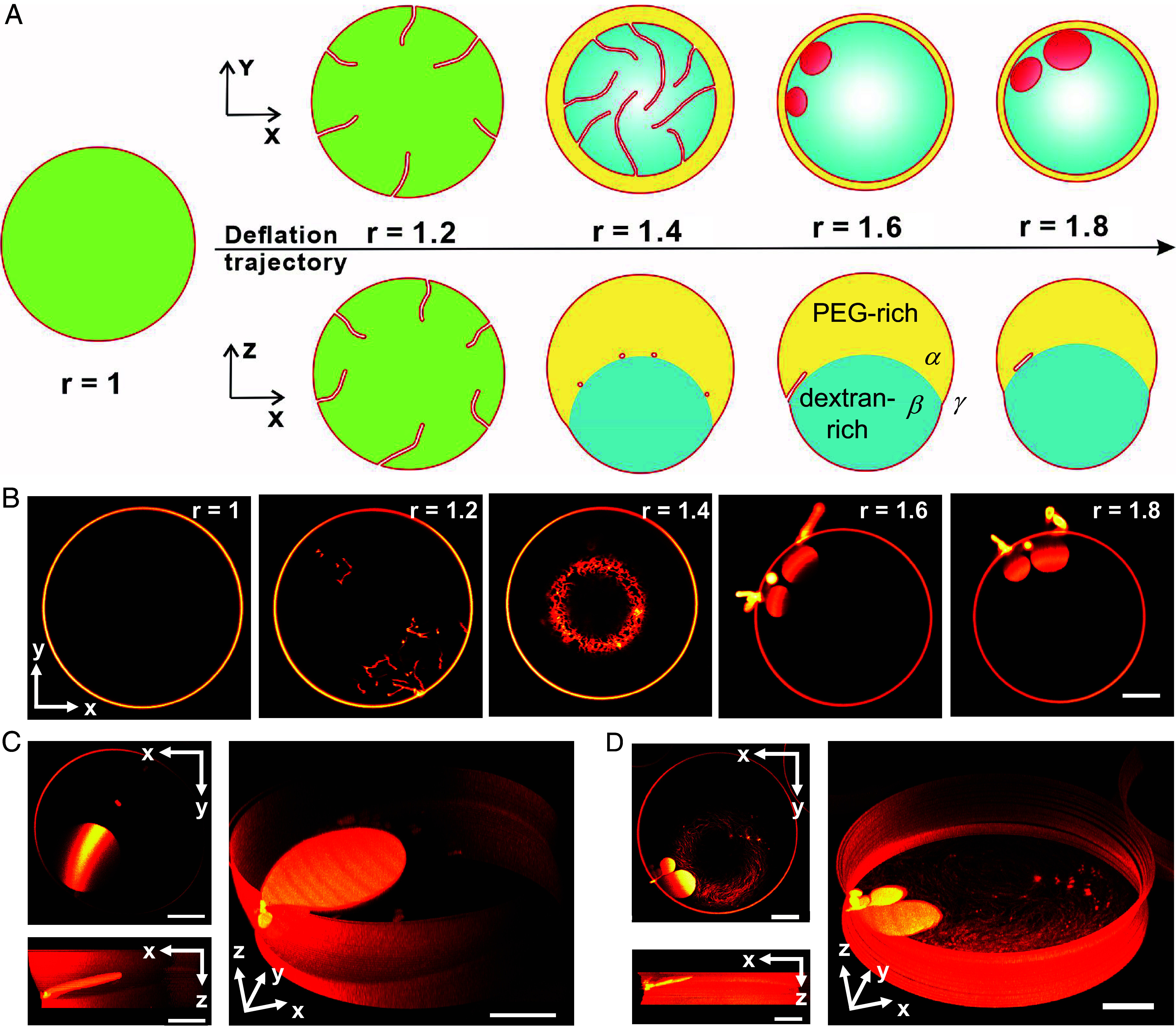
Tube-to-sheet transformation in a single GUV during deflation. (*A*) Morphological transformation of the vesicle-droplet system upon deflation shown schematically as xy projections (*Top* row) and vertical xz cross-sections (*Bottom* row). The osmolarity ratio r is characterized by the ratio between the exterior and the initial interior osmolarity. Before deflation (r = 1), the vesicle membrane (red) encloses a homogeneous (green) interior solution. Initial deflation triggers tube formation (not drawn to scale). Deflation above r = 1.2 causes bulk phase separation into a PEG-rich (*α*, yellow) and a dextran-rich (*β*, blue) phase transforming the vesicle into a Janus GUV. (*B*) Confocal xy cross-sections of the same vesicle monitored at different osmolarity ratios. Disk-like membrane sheets are observed for r = 1.6 and above. (*C* and *D*) 3D reconstructions of DMS from confocal z-stacks with a z-increment of 500 nm between stacks. Upper left images: xy-scans of sheets at the interface; *Lower Left* and *Right* images: side view and oblique side view of DMSs (i.e. disk-like vesicles) which follow the curvature of the *αβ* interface, and upon formation can either consume all nanotubes (*C*) or coexist with them (*D*). Note that in xy-scans, the nanotubes and membrane sheets are only partially visible because the scan moves across the spherical-cap of the *αβ* interface resulting in color intensity difference on the sheet structures. (Scale bars: 10 μm.)

Upon osmotic deflation, water is forced out of the vesicle to balance the osmolarity, causing a decrease in volume. Because the total membrane area is conserved, excess area is stored in nanotubes protruding into the vesicle interior. These nanotubes are stabilized by PEG asymmetry across the membrane and their thickness reflects the spontaneous curvature generated by this asymmetry (21, 30–32). We define the osmolarity ratio r as the ratio between the exterior osmolarity and the initial interior osmolarity and use this ratio to distinguish different states along the deflation trajectory in the phase diagram; see *SI Appendix*, Fig. S2. Fig. 1 *A* and *B* illustrates the transformation of a single vesicle tracked during the deflation process. For r = 1, the exterior osmolarity is equal to the initial interior osmolarity and the vesicle is spherical. For r = 1.2, excess membrane area induced by deflation becomes stored in the form of nanotubes which are free to move within the homogeneous vesicle interior. For r = 1.4, the interior solution crosses the binodal into the two-phase region (*SI Appendix*, Fig. S2), forming a PEG-rich *α* and a dextran-rich *β* droplet inside the GUV to produce a Janus vesicle. The nanotubes adhere to the *αβ* interface to lower the free energy of the vesicle-droplet system. Surprisingly, creating more excess area at higher deflation (r = 1.6) leads to shortening of some nanotubes by transforming them into sheet-like circular structures. These structures remain stable upon further deflation (r = 1.8) and can either consume all nanotubes or coexist with them (Fig. 1 *C* and *D*). In our particular system, these sheets are stabilized by the ATPS interface and bend along it (Fig. 1 *C* and *D* side views). Below we show that they represent DMS, i.e. very flat disk-like vesicles, connected to the mother GUV (Early confocal images of membrane sheets at the *αβ* interface were obtained by Yanhong Li during her PhD studies in November 2009). The tube-to-sheet transformation is observed for other lipid compositions as well; see *SI Appendix*, Fig. S3.

### Cisternae-Like DMS Structures Revealed by 2D and 3D STED.

**Fig. 2.**
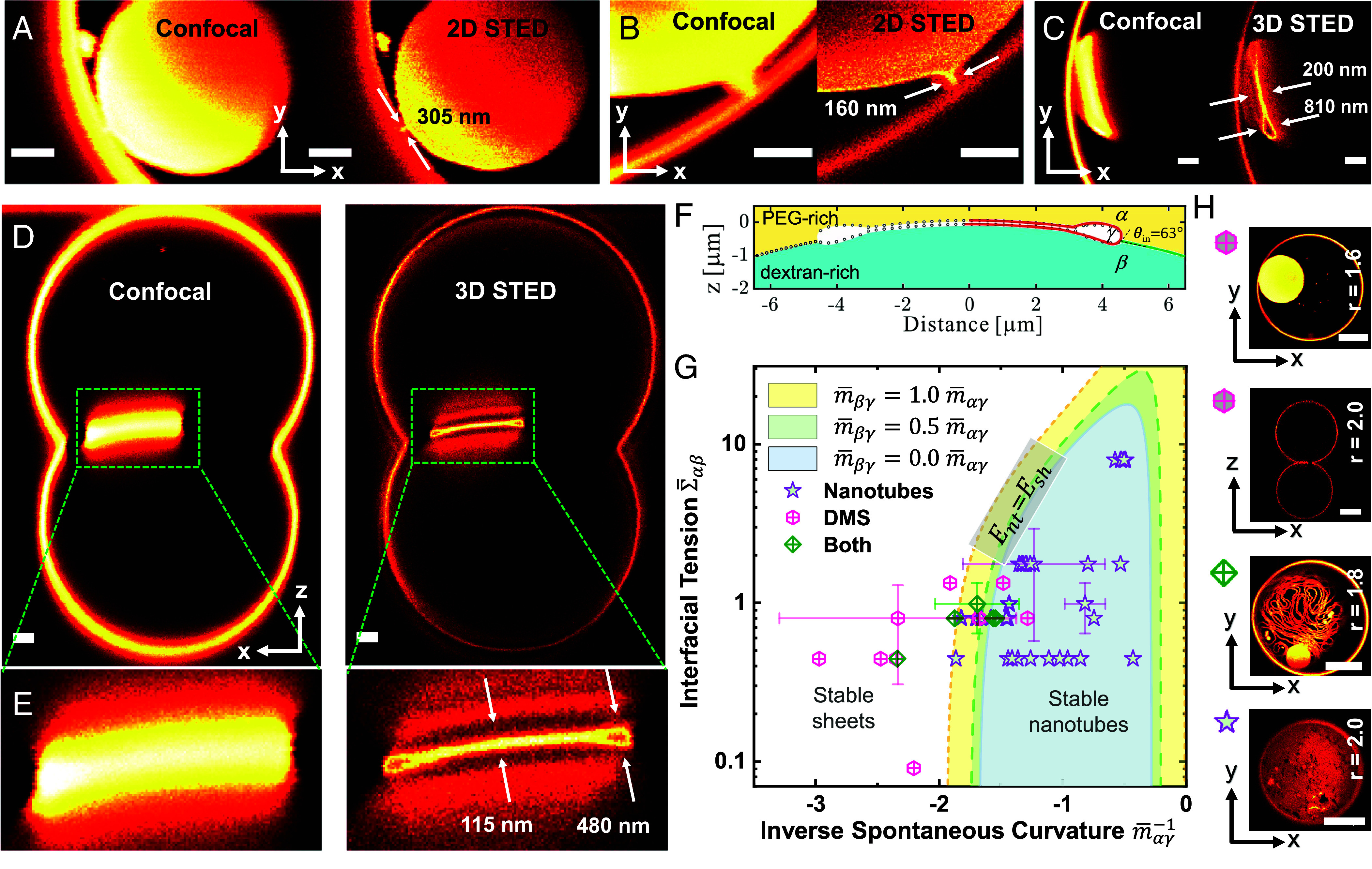
Cisternae-like DMS structure revealed with 2D and 3D STED and tube-to-sheet morphology diagram. (*A* and *B*) xy-scans of two DMS structures acquired by confocal and 2D STED with a pixel size of 50 nm (*A*) and 40 nm (*B*), selected to reduce artifacts from DMS movement during scanning of larger regions of interest. Membrane neck details are visualized with the superior lateral resolution from STED when a smaller region of interest is imaged as in *B*; see also *SI Appendix*, Fig. S4. The contour of the DMS appears sharper than that of the GUV membrane (*B*, *Right*) because the vesicle equator is away from the imaging xy-plane. (*C*) DMS xy-scans obtained with confocal and 3D STED with a pixel size of 50 nm. The improved axial resolution of 3D STED allows revealing the DMS cisternae-like structure and measuring its thickness. (*D*) Confocal and 3D STED xz-scans of the entire vesicle (r = 1.6) with a pixel size of 80 nm and (*E*) enlarged regions clearly confirming the double-membrane structure. (*A*–*D*) (Scale bars: 2 μm.) (*F*) The DMS profile measured with 3D STED (black dots) in Fig. 2*E* and numerically constructed DMS segments (red solid line) obtained by fitting circular arcs; the liquid–liquid interface is measured separately (solid green line on the right side of the DMS) and observed to have radius *R_αβ_* = 24 μm. (*G*) Morphology diagram constructed based on Eq. **2** and the model parameters from the circular arc fits in *F*; see *SI Appendix* for details and parameter values. The curves are transition lines at which the sheets and nanotubes have the same energy. Each transition line corresponds to a different spontaneous curvature of the *βγ* segment (see legend), for which no experimental values are available. In each shaded region bounded by one of the transition lines, the nanotubes provide the most stable morphologies whereas membrane sheets are the most stable structures outside this region. The data points (49 in total) represent the experimentally obtained values of the parameters. They were acquired for values of the interfacial tension $\Sigma_{\alpha \beta }$ varied in the range between 0.31 μN/m and 15.66 μN/m corresponding to different osmolarity ratios, and for three different membrane compositions, with the aim of exploring different condensate-to-membrane affinities and membrane bending rigidities; see *SI Appendix*, Fig. S7 for detailed descriptions and all error bars for the data points. The observed morphologies correspond well to the theoretically predicted regions. (*H*) Example images of GUVs from different regions of the morphology diagram for which the parameter values have been obtained. Each GUV is labeled with the same-colored symbol as used in the morphology diagram in *G*. (Scale bars: 10 μm.)

To resolve the DMS structure, we performed high-resolution STED imaging. To ease comparison and demonstrate the fine details unresolved by conventional microscopy, we provide STED images in Fig. 2 corresponding to the confocal scans. Each DMS is connected to the mother vesicle via a membrane neck (Fig. 2 *A* and *B*), serving as a link between the internal and external membrane areas. The membrane necks have a thickness of 305 nm and 160 nm in Fig. 2 *A* and *B* as determined from 2D STED line profile analysis (*SI Appendix*, Fig. S4), comparable to nanotube diameters in such systems (21, 31). Since the DMS is constantly moving along the three-phase contact line, and due to the small neck size, finer structures of the neck could not be resolved in Fig. 2*A*. However, scanning a smaller region with adequate scan speed improves STED resolution and reveals the smoothly curved membrane neck region (Fig. 2*B*). To resolve the DMS morphology (resembling a deflated erythrocyte), which is not feasible with confocal or 2D STED, we employed 3D STED with an axial resolution of about 110 nm, which drastically eliminates the out-of-focus signal. In Fig. 2*C*, the DMS lumen is revealed with an interstitial bilayer separation of about 200 nm in the middle and up to about 810 nm in the rim (note that the cross-section dimensions are affected by the DMS orientation at the curved interface with respect to the scanning plane and could appear enlarged). This shape is reminiscent of Golgi or ER cisternae, as observed by electron microscopy on ER in contact with the plasma membrane (33). The highly curved double-membrane structure in ER cisternae is shaped and maintained by luminal proteins (3, 7). In contrast, our system does not require any protein to maintain the DMS shape. A further 3D STED xz scan providing the side view of a Janus GUV (Fig. 2 *D* and *E*) confirms the cisterna-like double-membrane structure and the proper DMS orientation allows for a more precise measurement of the thicker rims (~480 nm) and thinner luminal gap in the middle (~115 nm); *SI Appendix*, Fig. S4 *D* and *E*. The dimensions of individual DMS vary based on the vesicle interior content.

### Theory of Morphological Nanotube-to-Sheet Transformation.

We construct a morphology diagram based on the cisternae-like structure resolved by STED. The cross-section profile of this structure at the *αβ* interface was constructed from the discrete DMS thickness and the curvature of the interface between the PEG-rich *α* and dextran-rich *β* phases (*R_αβ_* = 24 μm for the DMS in Fig. 2*D*), and the intrinsic contact angle $\theta_{\mathrm{in}}=63^{\circ }$ [obtained from the apparent contact angles by circular fitting (21)]. We fit five circular segments, labeled by *n* = 1,2,…,5, to construct the DMS surface and one circular segment to construct the *αβ* interface; see Fig. 2*F* (the fit parameters are given in *SI Appendix*, Tables S1 and S2). By integrating the line profiles obtained from the piece-wise circular fit to the experimental STED image with the theory of wetting (*Methods* and *SI Appendix*, Fig. S5), we can reliably estimate the parameter values for the nanotube-to-sheet transformation. The experimental observations provide evidence that the tube-to-sheet transformation proceeds in a local manner, without significant exchange of membrane area with the mother vesicle (see evidence in *SI Appendix*, Fig. S6). We can then focus on the membrane protrusions in the interfacial region. When the vesicle volume is reduced by osmotic deflation, the nanotubes first protrude into the PEG-rich *α* phase and then start to adhere to the *αβ* interface. Eventually, the adhering nanotubes are locally transformed into adhering membrane sheets. We now focus on the transformation of one nanotube. The total energy $E_{\mathrm{tot}}^{\mathrm{nt}}$ of the adhering nanotube and the total energy $E_{\mathrm{tot}}^{\mathrm{sh}}$ of the resulting membrane sheet can be obtained theoretically, where we impose the constraint that both types of protrusions have the same membrane area; see SI for details of the derivations. The adhering tubes are transformed into adhering sheets when the total energy of adhering membrane sheets is less than the total energy of adhering membrane nanotubes, $E_{\mathrm{tot}}^{\mathrm{sh}}\leq E_{\mathrm{tot}}^{\mathrm{nt}}$. The equality $E_{\mathrm{tot}}^{\mathrm{sh}}=E_{\mathrm{tot}}^{\mathrm{nt}}$ defines the transition point in terms of the critical interfacial tension $\Sigma_{\alpha \beta }^{*}$; see derivation of *SI Appendix*, Eq. **S2**. For interfacial tensions $\Sigma_{\alpha \beta }$ below the critical value $\Sigma_{\alpha \beta }^{*}$, membrane nanotubes are the stable morphology but for higher interfacial tensions with $\Sigma_{\alpha \beta }^{*}<\Sigma_{\alpha \beta }$, membrane sheets are energetically more favorable.

All parameters that determine the critical interfacial tension are either obtained from fits to the observed shape contour or from independent experimental measurements, for details see *SI Appendix*. Here, we assume that nanotubes have constant mean curvature which is measured from the nanotube diameter $D_{\mathrm{nt}}$ assessed with STED microscopy and spontaneous curvature which is assessed from the force balance $m_{\alpha \gamma }=-\sqrt{\left(\Sigma_{\alpha \beta }\sin\theta_{\beta }\right)/\left(2\kappa \sin\theta_{\gamma }\right)}$ along the contact line, where $\theta_{\beta }$ and $\theta_{\gamma }$ are apparent contact angles; see ref. 21. The membrane bending rigidity κ was measured via fluctuation analysis as explained in the *Methods* section. By taking the spontaneous curvature in the αγ segment (γ refers to the external aqueous solutions) as a free parameter and plotting the critical interfacial tension, we obtain the morphology diagram of the membrane tubes and sheets as a function of the rescaled interfacial tension ${{\bar{\Sigma }}_{\alpha \beta }=\Sigma }_{\alpha \beta }\left(D_{\mathrm{nt}}^{2}/\kappa \right)$ and the rescaled inverse spontaneous curvature ${{\bar{m}}_{\alpha \gamma }^{-1}=(1/m}_{\alpha \gamma }D_{\mathrm{nt}})$; see Fig. 2*G*. One parameter that has not been deduced from independent experiments is the spontaneous curvature $m_{\beta \gamma }$ of the *βγ* membrane segment. We take $m_{\beta \gamma }$ to be proportional to $m_{\alpha \gamma }$ (considering that at a certain deflation step, both membrane segments are wetted by the same external medium γ). Thus, the morphology diagram in Fig. 2*G* is presented as transition lines for different values of $m_{\beta \gamma }$, where the membrane sheet and the membrane nanotube have the same energy at each transition line; see *SI Appendix*, Fig. S7 for details. Our results indicate that the spontaneous curvature induced by the dextran-rich phase should be negligible. The morphology diagram includes different sets of experimental data points with nanotubes and membrane sheets (Fig. 2*H*) illustrating very good agreement with the theoretical model. Close to the transition lines, we observe coexistence of nanotubes and sheets, which implies that, in this region, both the nanotubes and the sheets are metastable states, separated by an energy barrier. In order to study these metastable states, one needs to determine the energy landscape and calculate the energy barrier for the transition from nanotube to sheet. For the complex rim geometry as depicted in Fig. 2 *D* and *F*, such a computation is very difficult. Therefore, we used a simpler toroidal rim geometry, for which the metastable nanotube state can be studied in detail; see Section on *Nanotube-Sheet Coexistence* and Fig. 5 further below.

The shape of the morphology diagram is not very sensitive to the intrinsic contact angle; see *SI Appendix*, Fig. S8 *A* and *B* where we display a few cases close to the original intrinsic contact angle in the system. In addition, we have considered i) two different cases, for which the spontaneous curvature of the *βγ* segment is positive, $m_{\beta \gamma }> >0$, see *SI Appendix*, Fig. S8*C*, and ii) another case, for which the spontaneous curvature of the nanotube is not taken to be uniform, see *SI Appendix*, Fig. S8*D*. The latter cases slightly shift the location of the transition lines and accommodate all experimental observations of DMS correctly in the stable sheet region of the morphology diagram or very close to the transition line. In addition, because the model only considers the energy of either nanotube or sheet, it does not elucidate the transformation pathway, which can be very complex as we show below.

### DMS Occurrence, Growth, Medium, and Lipid Exchange.

**Fig. 3.**
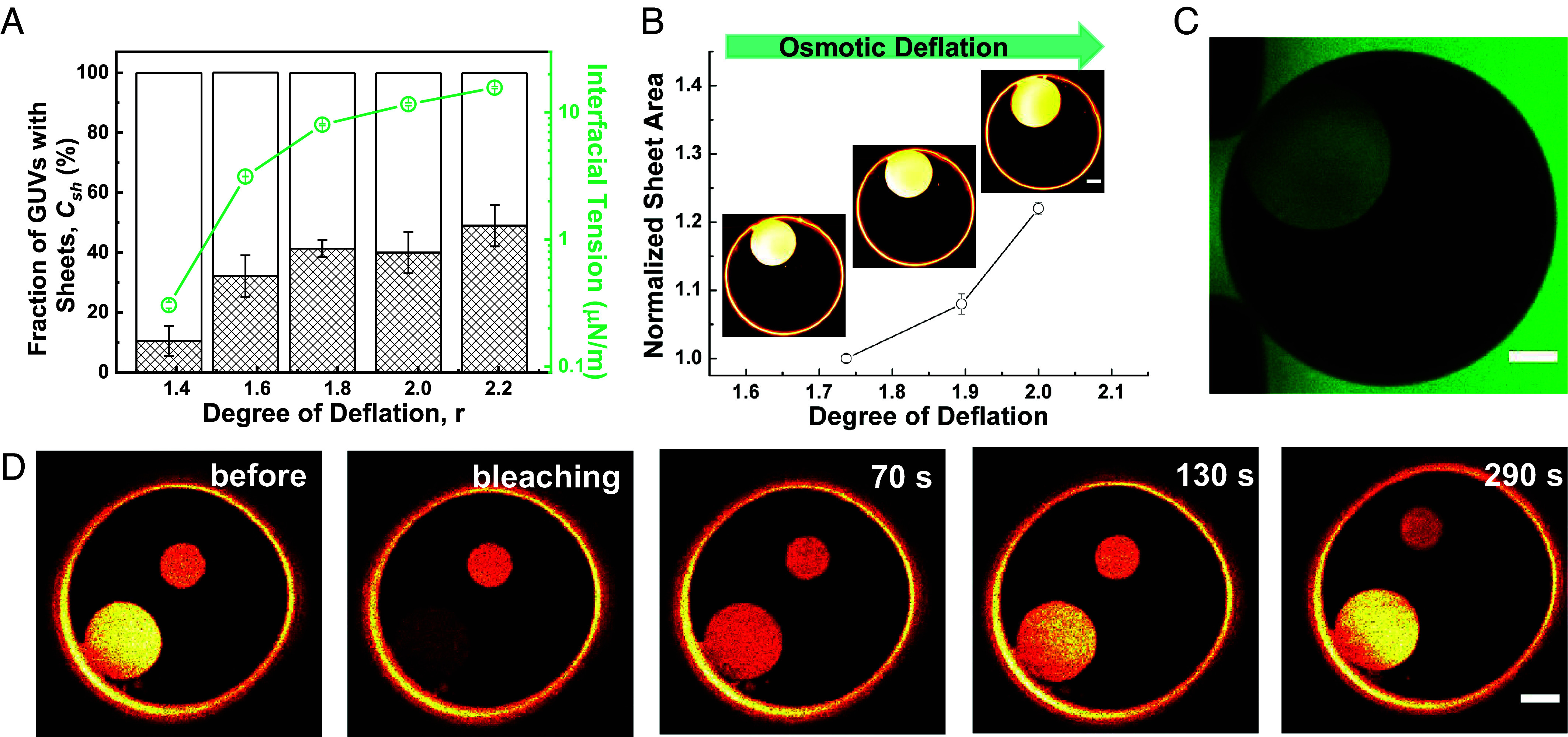
DMS occurrence, area growth, medium, and lipid exchange. (*A*) Fraction of GUVs having membrane sheets (bars) vs. osmolarity ratio r, and the corresponding interfacial tension (cyan open circles); see *SI Appendix*, Fig. S9 and Table S3 for details. (*B*) Normalized projected sheet area in the same vesicle showing a net increase of 8% and 14% with the last two steps of deflation. (*C*) Diffusion of the water-soluble and membrane-impermeable dye sulforhodamine B (green) from the external medium into the formed DMS through the neck: After DMS formation, the vesicle external solution was exchanged with medium containing 20 μM of sulforhodamine B. (*D*) FRAP images of a DMS at the ATPS interface with time stamps indicating the time after photobleaching the membrane dye; as a reference, a second smaller DMS (*Upper Middle* part of the image) is observed to exhibit roughly constant fluorescence signal. The fluorescence recovery curves of DMS and the corresponding basal vesicle membrane are shown in *SI Appendix*, Fig. S10. The fluorescence recovery time is affected by the neck size between the mother vesicle and the DMS and may vary for different GUVs. (Scale bars: 5 μm.)

To evaluate the fraction of GUVs exhibiting tube-to-sheet transformation, we explored different osmolarity ratios. GUVs with unresolvable defects (fluorescence puncta) or multilamellar structure were excluded from the analysis, accounting for 5 to 14% of the total number of GUVs. The investigated GUVs depicted in Fig. 3*A* were categorized into three distinct groups: vesicles with DMS, as shown by the hatched bars, and the remaining vesicles featuring only nanotubes (whether with and without knots) or exhibiting only budding. Between 200 and 400 GUVs were screened at each examined condition. As shown in Fig. 3*A*, at the first deflation step (r = 1.4) in the two-phase region, the percentage of GUVs having DMSs is the lowest, around 10.5%. This is reasonable considering the relatively small number of nanotubes in the system defined also by the small excess area created by deflation. With further deflations, we see an increasing number of GUVs having membrane sheets, reaching a plateau, which could result from nanotube obstruction by knots as discussed below. By increasing the osmolarity ratio r, the PEG–dextran interfacial tension $\Sigma_{\alpha \beta }$, measured independently, increases (Fig. 3*A*, cyan data; see *Methods* and *SI Appendix*, Fig. S9 and Table S3). This positively correlates with the increasing sheet fraction, indicating decrease of the total energy of the adhered membrane structures at the *αβ* interface, as the membrane sheet state becomes energetically more favorable (discussed in more detail in the next section).

To resolve to what extent the interior volume in the DMSs is connected to the external medium, we tracked DMS size change with deflation. DMS area grows (Fig. 3*B*) suggesting both lipid supply and medium exchange between the DMS and the mother vesicle. In some GUVs, we also observed DMS area decrease upon further deflation because of competition with area required for the vesicle budding. This depends on the initial volume-to-area ratio of the GUV and variations in the encapsulated polymers due to the large size [none of which are precisely controlled by the preparation protocol (34)]. To prove fluid exchange, water-soluble dye was perfused after DMS formation, Fig. 3*C*. The dye diffuses into the DMS, proving that the membrane neck is unobstructed and serves as a channel for medium exchange. Fluorescence recovery after photobleaching (FRAP) of the membrane dye (ATTO-647N-DOPE) in an entire DMS also shows gradual recovery, Fig. 3*D*, indicating exchange and supply of lipid molecules to the DMS from the mother vesicle via the neck. The half recovery time of the DMS is about 3 to 5 times longer than that of the basal mother vesicle membrane (*SI Appendix*, Fig. S10) because of retardation imposed by the neck.

### Tube-to-Sheet Transformation Pathways.

Before exploring the tube-to-sheet transformation pathways, we probed whether lipid oxidation, which is known to cause structural changes of the membrane (35–37) potentially induced by the strong laser irradiation, is the cause for DMS formation. Vesicle deflation was done in the bulk and observation was entirely performed in transmission light of low power (Movie S1). DMS formation was observed as expected, thus eliminating the possibility of tube-to-sheet transformation triggered by light-induced lipid oxidation.

**Fig. 4.**
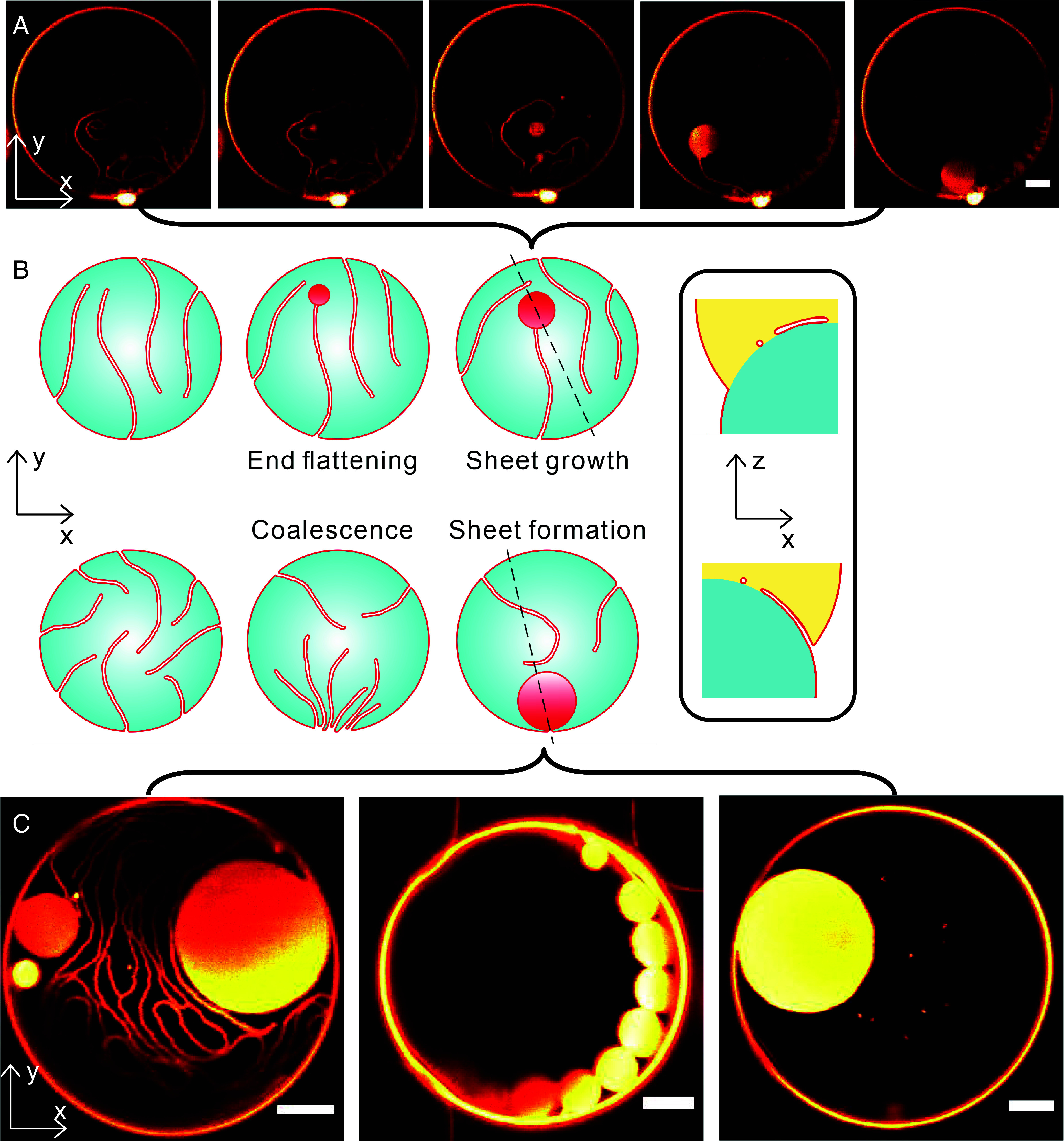
Two tube-to-sheet transformation pathways. (*A*) Time-lapse images of DMS forming from the end region of a nanotube and gradually growing back to the mother vesicle at the expense of shortening the nanotube. The time scale of the tube-to-sheet transformation process varies, the process presented here took about 109 s; see *SI Appendix*, Fig. S11 and Movie S3 for full sequence and more examples. (*B*) Schematic representation of two tube-to-sheet transformation pathways viewed from xy cross-sections at the interface of the corresponding GUV, with the *Top* row indicating tube-to-sheet formation initiating from the end of a nanotube and the *Bottom* row representing tube-to-sheet formation from coalescence of interfacial nanotubes originating at their orifices. Corresponding zoomed vertical (xz) cross-sections passing through the nanotube and membrane sheet are presented on the *Right*. (*C*) Examples for DMSs formed after nanotubes coalescence. Such DMSs are always located at the contact line and can coexist with nanotubes and other multiple DMSs. (Scale bars: 5 μm.)

Monitoring the tube-to-sheet transformation revealed two pathways. The first pathway is initiated from the end of a nanotube (Fig. 4*A*), which is highly curved and thus susceptible to shape changes to reduce the large adhesion energy (31, 38). The nanotube end either flattens into a disk, gradually increases its contact area with the ATPS interface (Movie S2) or first branches before transforming into a DMS (Fig. 4*A* and Movie S3), in both cases at the expense of shortening the nanotube (Fig. 4 *B*, *Top* row). Nanotube branching (initially via three-way junction) observed here was also shown in Monte Carlo simulations of tubes with increasing area-to-volume ratio (38) (occurring here via deflation). This end-flattening transformation process is relatively slow (minutes) and could be captured entirely (*SI Appendix*, Fig. S11 and Movie S3). It is worth noting that the transformation process varies in time scale, between a few minutes for complete transformation and roughly 10 min without obvious sheet growth/size change after spotting. In the second pathway (Fig. 4*C*), membrane sheets form by nanotube coalescence. The orifices of the nanotubes adsorbed at the interface diffuse around along the contact line on the mother vesicle surface and when approaching each other, presumably due to capillary forces, the merged neck opens and propagates into the vesicle interior until all adjacent nanotubes are used up to form a complete sheet structure (Fig. 4 *B*, *Bottom* row), the process is fast and takes only 1 to 2 s as witnessed by direct visual observation through the oculars. The transformation process can either consume all the nanotubes at the *αβ* interface or only a fraction of them, and either single or multiple DMSs can form depending on the number and location of the coalescing nanotubes. Once formed, the DMSs were unlikely to coalesce with another free nanotube or DMS as the relatively big size of the DMS prohibits the two membrane necks to meet and coalesce. The transformation process typically occurs in GUVs exhibiting a large number of nanotubes. For a higher level of deflation, the ATPS interface can be crowded with nanotubes, making the transformation process more likely in order to accommodate the excess membrane area. This could be one of the reasons for the increased sheet formation percentage at a higher osmolarity ratio (Fig. 3*A*). However, in some vesicles, we did witness both of the above-mentioned types of transformation (*SI Appendix*, Fig. S12). It is worth noting that the first transformation pathway is the dominant one based on our observation.

### Nanotube-Sheet Coexistence.

**Fig. 5.**
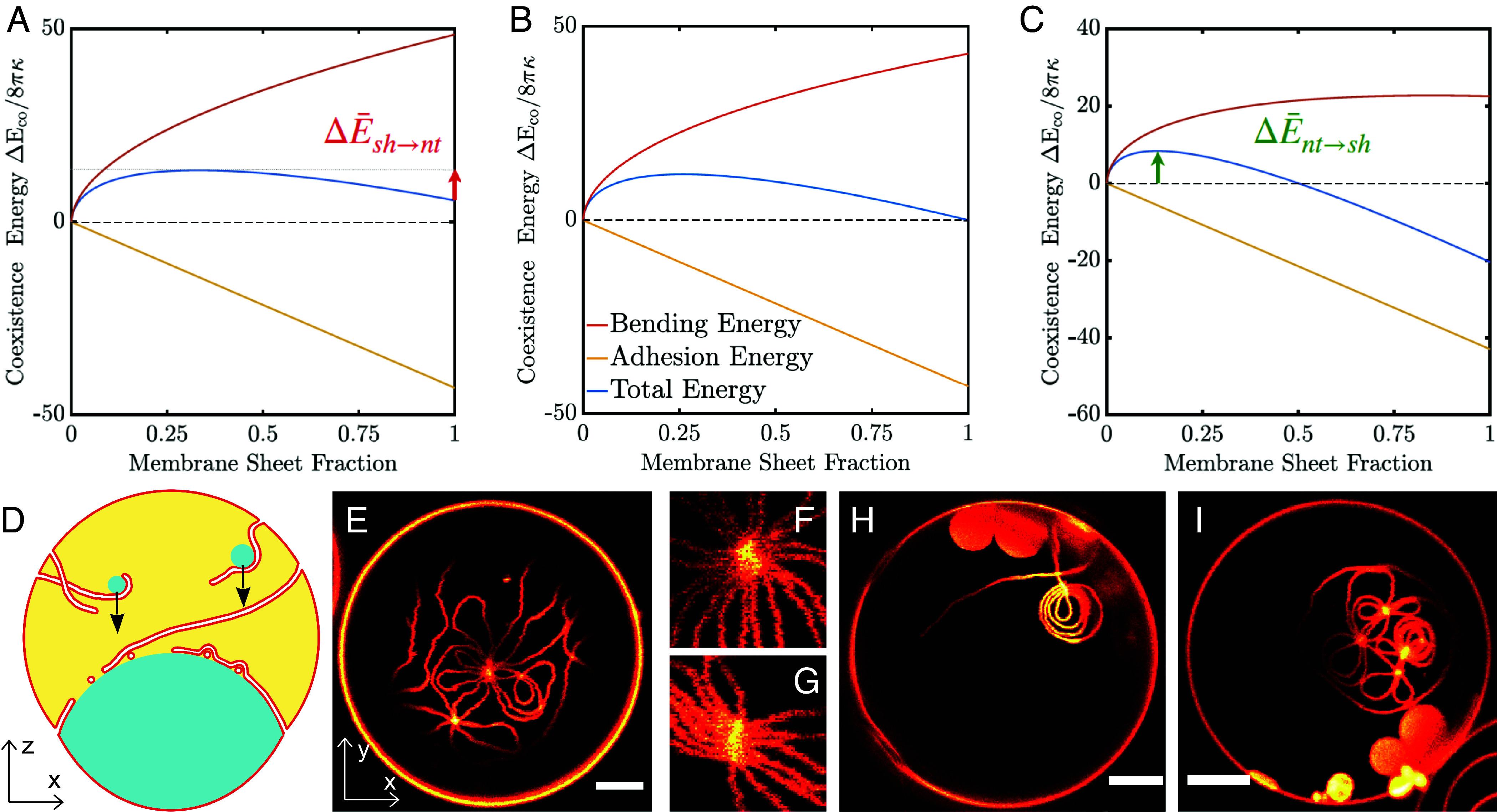
Energy landscape for different combinations of material parameters, and prohibition of DMS formation by nanotube intertwined knots. (*A*–*C*) The bending, adhesion, and total energy scaled by the bending energy of a sphere with zero spontaneous curvature $\Delta {\bar{E}}_{\mathrm{co}}=\Delta E_{\mathrm{co}}/8\pi \kappa$, are shown in red, yellow, and blue, respectively. Different coexistence energy conditions include (*A*) stable-nanotube/metastable-sheet *m* = −1/(186 nm), (*B*) stable-nanotube/stable-sheet *m* = −1/(186.84 nm), and (*C*) metastable-nanotube/stable-sheet, *m* = −1/(190 nm). For all cases both membrane segments and nanotube have the same spontaneous curvature *m_βγ_ = m_αγ_ = m_nt_*, the bending rigidity is *κ* = 14.9 [k_B_T] as measured in *SI Appendix*, Table S4, the interfacial tension is *Σ_αβ_* = 3.13 [μN/m] and the total membrane area *A* = 142.51 [µm^2^] which is obtained from the fit to experimental data in Fig. 2*E*. The red arrow in *A* shows the energy barrier of transformation from metastable-sheet to stable-nanotube, $\Delta {\bar{E}}_{sh\to nt}$ and the green arrow in *C* shows the energy barrier of transformation from metastable-nanotube to stable sheet $\Delta {\bar{E}}_{nt\to sh}$, respectively. (*D*) Schematic illustration of how nanotubes plausibly entangle during formation, while adhering to sedimenting droplets (that form from ongoing phase separation in the PEG-rich phase upon vesicle deflation) and upon settling to and adhering at the two-phase interface one over another, resulting in nanotube knots. (*E*) A single nanotube forms several knots with itself, which hinders further transformations. (*F* and *G*) Two example images (4 μm × 4 μm) for nanotube knots acquired with STED at a pixel size of 50 nm; the "aster" and "bundle" shaped nanotubes are tightly bounded together and move like one single structure; see Movies S5–S7. (*H* and *I*) Exemplary images of GUVs with DMSs coexisting with nanotube knots at the interface. The knots hinder the lipid diffusion and water influx in the specific nanotube thus prohibiting its further shape transformation. (Scale bars: 5 μm.)

Our study suggests that the nanotube and sheet structures are very soft and flexible, they can reversibly transform into one another and coexist (Movies S2–S4). To understand this feature of the system, we developed a nanotube-sheet coexistence model for fixed membrane area accommodated at the PEG–dextran interface; see *SI Appendix* for more details. We define the coexistence energy $\Delta E_{\mathrm{co}}$ which measures the energy difference between the state of coexisting nanotubes/sheets and the state where only nanotubes are present. In Fig. 5 *A*–*C*, several examples of the coexistence energy, are plotted as a function of the membrane sheet fraction for various spontaneous curvatures close to the transition lines. We can distinguish different examples of nanotube-sheet coexistence by different stability conditions. For instance, for spontaneous curvature *m* = −1/(186 nm), the membrane nanotubes are in the stable state (zero membrane sheet fraction stands for the state where the total area is stored in nanotubes only); and as we increase the membrane sheet contribution, the total energy of the interface increases and leads to metastable membrane sheets, which are separated by an energy barrier $\Delta {\bar{E}}_{sh\to nt}=7.91$ shown by the red arrow in Fig. 5*A*. If we slightly increase the spontaneous curvature to *m* = −1/(186.84 nm), we obtain a bistable state at the transition line where both nanotubes and sheets are stable states; see Fig. 5*B*. In addition, if we slightly increase the spontaneous curvature further to *m* = −1/(190 nm), the energy of the nanotube state exceeds the energy of the sheet state and both states are separated by an energy barrier $\Delta {\bar{E}}_{nt\to sh}=8.39$ (green arrow in Fig. 5*C*). These examples demonstrate that stability conditions can change in a very narrow window of parameter space close to the transition lines, here spontaneous curvature being the control parameter. Note that such small changes of the spontaneous curvature can be provided simply as a result of thermal fluctuations. The SD associated with material parameters such as the spontaneous curvature in DMS (Fig. 2*G*) and nanotubes (21) provide the basis for this claim.

### Nanotube Knots Prohibit Tube-to-Sheet Transformation.

After resolving the origin of DMSs, we were curious why other GUVs do not have DMSs inside; have they reached an equilibrium state? The situation varies but mostly related to how nanotubes become accommodated at the *αβ* interface. Nanotubes start forming during the first deflation step, fluctuating and exploring the entire vesicle interior. After the second deflation, nanotubes start adhering at the formed two-phase interface confining fluctuations to a quasi-two-dimensional surface (as sketched in Fig. 1*A*). As phase separation proceeds with further deflation, droplets of dextran-rich phase form within the PEG-rich one. Upon encounter, tubes in the PEG-rich phase adhere or wrap around droplets which drag them down settling onto the interface, occasionally crossing and piling onto other previously adsorbed nanotubes or entangling with others in the bulk (Fig. 5*D*). With even further deflation, nanotubes can elongate and new nanotubes can nucleate, preferentially from the PEG-rich phase due to PEG asymmetry across the membrane (31, 32). Upon getting accommodated onto the two-phase interface and depending on how crowded it is, new tubes may entangle and “lock” a number of other nanotubes already at the interface, producing aster- or bundle-like projections (Fig. 5 *E*–*I*), which we define as nanotube knots. The nanotube entanglement is fairly strong and knots seem to behave as one integrated structure (Fig. 5 *F* and *G* and Movies S5–S7). Additionally, 3D STED xzt-scans (Movie S8) show that all nanotubes at the knot site are bounded tightly and potentially squeezed, thereby hindering lipid diffusion and water influx into the nanotubes and suppressing further transformation into DMS. Further evidence is shown in Fig. 5 *H* and *I* where we see DMSs coexisting with nanotubes forming knots with themselves and remaining kinetically trapped. This explains the saturation of DMS formation at higher deflation levels in Fig. 3*A*. A small fraction of the DMS-free vesicles exhibited nanotubes that were not entangled by knots, which we predominantly attribute to the nonuniform polymer encapsulation in the different vesicles (39, 40) and their initial tension (see *SI Appendix* for details). Knots are observed more frequently with longer/more nanotubes, both of which are modulated by deflations. Membrane tubule contact is ubiquitous in eukaryotic cells, it was shown that ER tubules may entangle with mitochondria (membrane-bound organelles) fission sites mediating membrane transformation ultimately leading to mitochondrial division (41, 42). The nanotube knots in our study are reminiscent of this complex network of membrane tubules and could potentially indicate further nanotube elongation and fission.

### Relevance to Biological Organelles.

Liquid–liquid phase separation is a common feature in cells, with biomolecular condensates exhibiting a wide range of interfacial tensions (43), including those explored in this study. Given that the ER network spans over the entire cell, it is highly probable that the ER membrane interacts with biomolecular condensates within the cell, as evidenced by several studies (9–12, 44, 45). Other similar double-membrane structures, such as phagophores (cup-like double-membrane structures) and autophagosomes (double-membrane vesicles), also exhibit membrane transformations. The initiation of phagophores from ER subdomains requires the participation of Atg family proteins (46, 47) (the complex roles of each protein are still under investigation), while the closure of DMS into autophagosomes, devoid of most transmembrane proteins (48, 49), may involve wetting-mediated membrane transformation, with ESCRT machinery facilitating final pore closure via fission (49, 50). Indeed, emerging evidence points to the involvement of biomolecular condensates in the process of autophagosome formation (51–53).

Our research on model membranes coupled with liquid–liquid phase separation aims to provide insights and expand the spectrum of membrane transformation phenomena induced by condensates. Previous studies have shown that these interactions can lead to membrane ruffling and fingering (54), lipid condensation (55), and even endocytosis-like processes (56, 57), as reviewed elsewhere (58). Here, we demonstrate that condensate wetting and membrane area changes can lead to interconversion between the two highly curved membrane structures (tubes and DMSs). Our findings suggest that the ER membrane could be reshaped by the wetting behavior of biomolecular condensates in cells, albeit in synergy with transmembrane proteins playing significant and active roles. Further meticulously designed studies are needed to fully understand this phenomenon, potentially benefiting the biophysics community.

## Conclusion

In this work, we observed the formation of DMSs and tube-to-sheet transformation in lipid vesicles enclosing condensates. The nanoscopic structures revealed by 2D and 3D STED resemble characteristic elements of the ER, namely, cisternae and tubules. We identified two tube-to-sheet transformation pathways and used theoretical analysis to obtain morphology diagram of the tube-to-sheet transformation. Overall, compared to nanotubes, DMSs provide a much more efficient way of storing membrane area. In extreme conditions, the tube-to-sheet and the reverse transformations can provide ways for cells to survive extreme external osmotic shock. Based on our findings, tubular to cisternae-like structure transformation in the ER could also occur via membrane wetting by biomacromolecular condensates. The interfacial tension of the condensates, modulated by environmental conditions, as well as the variations of the membrane spontaneous curvature regulated by proteins present very plausible mechanisms for controlling the morphologies of various membrane-bound and membraneless organelles. Biomembranes undergo various shape transformations such as endocytic or exocytic budding as well as nanotube and membrane sheet generation during different stages of cellular events. Understanding the coexistence of nanotubes and DMSs as in the ER, the tube-to-sheet transformation, and the prohibition mechanisms based on tubular knots can shed light on the principles of similar cellular activities such as the evolution of ER cisternae and the replication of coronavirus at ER membrane compartments. In general, the tube-to-sheet transformations may deepen our understanding of how cells redistribute membrane upon wetting by internal biomolecular condensates.

## Methods

### Materials.

Dextran from Leuconostoc spp (Mr 450 to 650 kg mol^−1^, batch number: BCBR8689V), and PEG (PEG 8000, Mv 8 kg mol^−1^, batch number: MKBT7461V) were purchased from Sigma-Aldrich. 1, 2-dioleoyl-sn-glycero-3-phosphocholine (DOPC) and 1, 2-dipalmitoyl-sn-glycero-3-phosphocholine (DPPC) were purchased from Avanti Polar Lipids and cholesterol from Sigma-Aldrich. 1, 2-Dioleoyl-sn-glycero-3-phosphoethanolamine labeled with ATTO 647N (ATTO-647N-DOPE product number AD 647N-161, excitation peak at 646 nm, emission peak at 664 nm) was purchased from ATTO-TEC GmbH. All other reagents were of analytical grade. All solutions were prepared using ultrapure water from the SG water purification system (Ultrapure Integra UV plus, SG Wasseraufbereitung) with a resistivity of 18.2 MΩ.cm.

### Binodal, Critical Point, Density, and Osmolarity of ATPS.

The binodal of ATPS was determined from cloud point titration as described in details before (59, 60). At the critical point, the volumes of the two phases are equal as one approaches the binodal from the two-phase region. The critical point was determined by a reversed process of cloud point titration. The osmolarities of the mixed polymer solutions without and with sucrose of increasing concentrations were measured by an osmometer (Gonotec Osmomat 3000 Freezing point osmometer). The densities of these solutions were measured by a densitometer (Anton Paar DMA 5000M) and solution compositions selected to ensure that the Janus GUVs encapsulating ATPS always stand vertically on the coverslip to facilitate imaging.

### Vesicle Preparation and Deflation.

The GUVs were prepared with the conventional electroformation method as described previously (31, 61), from the ternary lipid mixture of DOPC:DPPC:cholesterol = 64:15:21 (mole fractions) in the Ld phase, and labeled with 0.5 mol% ATTO-647N-DOPE for STED super-resolution imaging. GUVs made of DOPC or DOPC:DPPC:cholesterol = 35:35:30 (exhibiting liquid ordered and liquid disordered Ld/Lo phase coexistence) were also explored for DMS formation. In addition to the Ld GUVs, we also tested two lipid mixtures in the Lo phase with higher bending rigidities, with Lo1 of DOPC:DPPC:cholesterol = 13:44:43, and Lo2 of DOPC:DPPC:cholesterol = 12:33:55. The GUVs encapsulated dextran/PEG ATPS in the one-phase region with a weight ratio of D/P = 1.57 (4.76% and 3.03% weight fractions), chosen so that subsequent hypertonic vesicle deflation would cross the critical point after reaching the two-phase region, thus resulting in similar volumes of the PEG-rich and dextran-rich phases (21). The lipid mixture in chloroform was deposited on two oppositely facing indium tin oxide-coated glasses and an electric field of 1.0 V_pp_ and 10 Hz was applied by a function generator for 2 h. Afterward, the GUVs were collected and used immediately. The vesicles were trapped and immobilized in a microfluidic device (21, 29) using a high-precision NeMESYS syringe pump. Once sufficient amount of GUVs were trapped by the posts of the microfluidic chip, deflation was initiated using dextran/PEG solution of D/P = 1 (3.54%, 3.54% weight fractions) with ~20% stepwise increase in osmolarity using sucrose. This choice of solutions ensures that the density of the external medium is between those of the upper and lighter PEG-rich and the lower and heavier dextran-rich phases. Typically, 10 to 15 times of the device volume (circa 4 μL) was exchanged by the deflation medium to ensure a complete process. The vesicles were then given time to equilibrate, and microscopy imaging was initiated.

### Microfluidic Chip Fabrication for Vesicle Immobilization.

The microfluidic device, as reported in ref. 21, has 8 flow channels each containing 17 vesicle traps; see *SI Appendix*, Fig. S1. The channels are equipped with fluid guiding posts for collecting vesicles. The device was fabricated by pouring degassed polydimethylsiloxane (PDMS) precursor and curing agent (Sylgard 184, Dow Corning GmbH), with a mass ratio of 10:1, onto a silicon wafer and baking at 80 °C for 2 h. PDMS block was gently peeled off from the wafer and cut into pieces, with inlet and outlet holes punched with a biopsy punch (Kai Medical). Glass coverslips were cleaned sequentially by detergent, water, and ethanol, blow dried with nitrogen, and baked at 100 °C. The PDMS device and coverslip were plasma-treated for 1 min using high-power expanded plasma cleaner (Harrick Plasma) before bonding. The device was then baked at 80 °C for 30 min to accelerate the bonding process. Solution exchange in the microfluidic device was controlled by a NeMESYS syringe pump using a 0.5 mL Hamilton gas-tight syringe. Before experiment, desired amount of solution was filled into the microfluidic device by centrifugation at 900 relative centrifugal force (Rotina 420R, Hettich). GUVs were typically collected in the traps at a flow rate of 1 μL/min, and STED imaging was conducted at an ultraslow flow rate of 0.035 μL/min, sufficient to keep the GUVs in the trap without imposing significant stress to the vesicle membrane. An image after GUV collection can be viewed in *SI Appendix*, Fig. S1*D*.

### STED Imaging.

The STED microscope from Abberior Instruments GmbH is based on an inverted microscope (IX83, Olympus Inc., Japan) equipped with a pulsed STED laser beam at 775 nm. To ensure STED functions with maximum resolution, alignment of the excitation and depletion beams was done first by inspection of the depletion focus shape in reflection mode using 150 nm gold beads (Abberior Nanoparticle Set for Expert Line 595 & 775 nm, Item number: AS-595-775-NP). Mismatches between the reflection mode and the fluorescence mode were corrected by imaging TetraSpeck beads of four colors (TetraSpeck™ Microspheres, 0.1 µm, fluorescent blue/green/orange/dark red). STED lateral resolution was measured using crimson beads of 26 nm diameter (Carboxylate-Modified Microspheres, FluoSpheres™, Molecular Probe) and found to be <40 nm (21). A spatial light modulator is used for generating 2D and 3D STED laser beam patterns, and 3D STED can greatly eliminate the out-of-focus signal in the axial axis thus improving the z-resolution to ~110 nm. GUVs were imaged on the STED expert line with 60× Olympus UPlanSApo water immersion objective (N.A. = 1.20).

Since the DMSs are constantly moving along the three-phase contact line, and due to its small size, finer structures of the neck could not be acquired in Fig. 2*A*. Since the DMS are curved (as is the two-phase interface to which they adhere) and in order to obtain higher spatial resolution, we performed 3D STED. Compared to confocal and 2D STED imaging, the xy focal plane thickness offered by 3D STED in our setup is greatly decreased to about 110 nm, which helps to eliminate the majority of the out-of-focus signal.

### FRAP.

FRAP experiments were performed with a Leica TCS SP8 (Wetzlar, Germany) microscope using HC PL APO CS2 63× (N.A. = 1.20) water immersion objective at 1 Airy unit. GUVs were trapped in the same microfluidic setup as in STED experiments to facilitate vesicle immobilization for FRAP. Imaging and photobleaching were performed with a 633 nm HeNe laser. Images were acquired with 256 × 256 format at a speed of 1,400 Hz (The confocal imaging series in Fig. 4*A* and *SI Appendix*, Fig. S11*B* were also acquired with the same imaging conditions to follow the transient and subtle changes of the membrane). Ten prebleach images at low laser intensity were recorded as a reference, then the laser intensity was increased to maximum for multiple frames in order to photobleach the DMS to almost zero intensity, after which the laser intensity was again decreased to record the fluorescence recovery behavior of the same membrane structure. FRAP curve was analyzed using commercialized Leica software. Data were fitted by an exponential model $y\left(t\right)=A\left(1-e^{-\tau t}\right)$, where *A* corresponds to the plateau intensity, *τ* stands for fitted parameter, and *t* is the time after bleach. The half recovery time *t*_1/2_ can be obtained using the following equation: $t_{1/2}=-\frac{\ln0.5}{\tau }$.

### Interfacial Tension Measurement.

The interfacial tension between the coexisting dextran-rich and PEG-rich phases was measured by a SITE100 spinning drop tensiometer (Krüss). Approximately 1 μL of the PEG-rich droplet was injected into a transparent glass capillary that was prefilled with bubble-free denser solution of the dextran-rich phase. The horizontally aligned capillary rotated at a certain speed *ω* between 500 and 12,000 rpm, and the lighter droplet became elongated along the axis of rotation. The interfacial tension, *σ*, between the two phases was calculated based on the Vonnegut equation at a sufficiently high rotation speed when the length of the droplet exceeded 4 times its equatorial diameter, 2*R_drop_*. The equation has the form $\sigma =\Delta \rho \omega^{2}R_{\mathrm{drop}}^{3}/4$, where *Δρ* is the density difference between the coexisting phases as measured by a densitometer. The radius *R_drop_* was measured directly in the commercial software by calibrating the pixel size using a stiff cylindrical stick with a known diameter in the capillary filled with the same solution of the dextran-rich phase. The interfacial tensions at different dextran and PEG weight fractions are listed in *SI Appendix*, Table S3.

### Fluctuation Spectroscopy.

Fluctuation analysis was performed on Ld vesicles encapsulating ATPS in the one-phase region following a protocol established earlier (62, 63). In short, the electroformed GUVs containing the polymer solution (of low-osmolarity ~19 to 20 mOsm/kg) were deflated by adding 2.5 to 5 mM sucrose to the external solution. The observation chamber was immediately sealed to prevent further vesicle deflation and binodal crossing due to evaporation. Fluctuation data were acquired at room temperature (~23 °C) on an inverted microscope (Zeiss Observer.D1) using a 40×/0.65 objective in phase contrast mode. A total of 1,800 images at the equatorial plane of each GUV were acquired by a high-resolution camera (pco.edge, PCO AG, Kelheim, Germany) with 200 μs exposure time at an acquisition speed of 15 frames per second. The GUV contour detection and analysis were performed using custom-built software as previously reported (62). Only GUVs with a clear contour and without inclusions were analyzed. The obtained bending rigidity data are summarized in *SI Appendix*, Table S4 and used for the theoretical calculations. The bending rigidities of Lo GUVs were approximated from literature values (64) as follows: for Lo1 membranes *κ* ≈ 83.4 [k_B_T] and for Lo2 membrane *κ* ≈ 53.7 [k_B_T].

### A Continuum Model of Membrane Wetting.

The total energy of the membrane which encapsulates two liquid phases of PEG-rich (*α* phase) and dextran-rich (*β* phase) can be divided into noninterfacial and interfacial terms. The noninterfacial energy of the system corresponds to the membrane bending energy at the cap regions, $E_{\mathrm{cap}}$, of the mother vesicle far from the *αβ* interface. The interfacial terms describe the energy of membrane protrusions, $E_{\mathrm{pr}}$, that adhere to the liquid–liquid (*αβ*) interface. Thus, the total energy (or shape functional) of the partially wetted vesicle has the form

[1] $$\begin{array}{c} E=E_{\mathrm{cap}}+E_{\mathrm{pr}}=\sum_{i=\alpha ,\beta }\left[\int {\mathrm{dA}}_{i\gamma }\left[2\kappa_{i\gamma }{\left(M_{i\gamma }-m_{i\gamma }\right)}^{2}\right]+\Sigma_{i\gamma }A_{i\gamma }-P_{i}V_{i}\right]\\ \;+2\kappa_{\mathrm{pr}}\int {\mathrm{dA}}_{\mathrm{pr}}\left[{\left(M_{\mathrm{pr}}-m_{\mathrm{pr}}\right)}^{2}\right]+\Sigma_{\alpha \beta }A_{\alpha \beta }, \end{array}$$

with the subscript *γ* referring to the external aqueous solution (Figs. 1*A* and 2*F*). The noninterfacial energy *E_cap_*, which is the sum over the two membrane segments with areas *A_αγ_* and *A_βγ_*, includes the bending energies of the respective membrane segments. The two membrane segment areas and the volume of the aqueous phases are conserved by employing four Lagrange multipliers, *Σ_iγ_* and *P_i_* with *i* = *α*, *β*, respectively. These membrane segments can, in general, have different bending rigidities *κ_αγ_* and *κ_βγ_* and different spontaneous curvatures *m_αγ_* and *m_βγ_*. The local mean curvature of the membrane is denoted by *M_iγ_*. The next two terms in Eq. **1** are the bending energy of the membrane protrusion as well as the interfacial free energy of *αβ* interface. The total membrane area and the total vesicle volume are constant, i.e., *A_me_ = A_αγ_ + A_βγ_ + A_pr_* and *V_ve_ = V_α_ + V_β_*, respectively. The bending rigidity is taken to be the same in the two membrane segments *κ = κ_αγ_ = κ_βγ_ = κ_pr_*, also the line tension of the three-phase contact line is assumed to be negligible *λ_αβγ_* = 0. A similar theoretical description of the membrane-droplet system has been introduced before (65, 66).

The total energy of the membrane protrusions is the sum of adhesion energy $E_{\mathrm{ad}}$ and bending energy $E_{\mathrm{be}}$. The morphological transformation from nanotube to DMS requires that the total energy of DMS wetting of the *αβ* interface is less than the energy of the membrane nanotubes $E_{\mathrm{ad}}^{\mathrm{sh}}+E_{\mathrm{be}}^{\mathrm{sh}}<E_{\mathrm{ad}}^{\mathrm{nt}}+E_{\mathrm{be}}^{\mathrm{nt}}$, and at a transition line, the energies of interfacial membrane nanotubes and sheets are equal $E_{\mathrm{tot}}^{\mathrm{sh}}=E_{\mathrm{tot}}^{\mathrm{nt}}$. This energetic criterion leads to the critical interfacial tension $\Sigma_{\alpha \beta }^{*}$ condition

[2] $$\begin{array}{c} \Sigma_{\alpha \beta }^{*}\equiv \frac{\pi }{A_{\mathrm{pr}}}\left[\frac{E_{\mathrm{be}}^{\mathrm{sh}}-E_{\mathrm{be}}^{\mathrm{nt}}}{\left(\theta_{\mathrm{in}}-\pi f_{\beta \gamma }\right)\cos\;\theta_{\mathrm{in}}-\Phi \;\cot\;\Phi \;\sin\;\theta_{\mathrm{in}}+\pi f_{\alpha \beta }}\right], \end{array}$$

where $A_{\mathrm{pr}}=A_{\mathrm{nt}}=A_{\mathrm{sh}}$ and the two area fractions are defined as ${f_{\beta \gamma }=A}_{\beta \gamma }/A_{\mathrm{pr}}$ and ${f_{\alpha \beta }=\Delta A}_{\alpha \beta }/A_{\mathrm{pr}}$. The detailed derivation of the Eq. **2** is provided in *SI Appendix* and all the parameters used to calculate the critical interfacial tension are tabulated in *SI Appendix*, Tables S1 and S2.

## Supplementary Material

Appendix 01 (PDF)

Movie S1. **DMS at ATPS interface viewed by transmission light microscopy.** ATPS GUVs (with 0.5% ATTO 647N DOPE) were diluted and deflated directly with 5~7 mM sucrose (r is between 1.3 and 1.4) added to the initial isotonic solution and screened for DMS formation. The movie was taken in phase contrast mode with a PCO camera (pco.edge) and a 40× /NA 0.65 objective at a frame rate of 20 fps on an inverted microscope (Zeiss Observer.D1) after vesicle incubation in a hypertonic solution. Manual adjustment of the focus in Z axis was needed in order to display the DMS which appears as a dense black circle (because of the refractive index difference between the two phases and external medium) at the ATPS interface. The PEG-rich and dextran-rich phase each appears as an optical light phase (top) and an optical dense phase (bottom). The movie is played with a frame rate of 50 fps. Scale bar is 5 μm.

Movie S2. **Tube-to-sheet transformation via end region flattening of the nanotube.** The movie displays the whole tube-to-sheet transformation process initiated from end region flattening of the nanotube which took about 11 seconds. Movie S2 corresponds to the GUV in Fig. 4a. The movie is played with a frame rate of 100 fps.

Movie S3. **Tube-to-sheet transformation via nanotube branching.** The movie displays the whole tube-to-sheet transformation process initiated from nanotube branching which took about 110 seconds. Movie S3 corresponds to the GUV in Fig. 4a. The movie is played with a frame rate of 100 fps.

Movie S4. **Membrane sheet-to-nanotube back transformation trajectory.** The movie displays the whole sheet-to-tube transformation process which took about 150 seconds. Movie S4 corresponds to the GUV in Fig. 4a. The movie is played with a frame rate of 100 fps.

Movie S5. **2D STED xy-t scans of nanotube knots.** Two exemplary nanotube knots movies captured by 2D STED, movie S5 features an aster-like knot and movie S6 features a bundle-like knot, both structures were tightly bounded together and could not disassociate, the bounded nanotubes are unlikely to go through further shape transformations. Movie S5 and S6 correspond to nanotube knots in Fig. 5f and 5g. The movies are played with a frame rate of 15 fps.

Movie S6. **2D STED xy-t scans of nanotube knots.** Two exemplary nanotube knots movies captured by 2D STED, movie S5 features an aster-like knot and movie S6 features a bundle-like knot, both structures were tightly bounded together and could not disassociate, the bounded nanotubes are unlikely to go through further shape transformations. Movie S5 and S6 correspond to nanotube knots in Fig. 5f and 5g. The movies are played with a frame rate of 15 fps.

Movie S7. **Enlarged nanotube knot video for Figure 5h.** The enlarged STED xy-t scan shows nanotube fluctuates on the interface while keeping the nanotube knot shape intact. The movie is played with a frame rate of 5 fps.

Movie S8. **Confocal and 3D STED xz-t scans of nanotube knots.** A thin layer of nanotubes is accompanied by a large knot in the center of the frame. Fine structures of the nanotube knots cannot be obtained by 3D STED indicating the gap dimension between the nanotubes is below 3D STED axial resolution which is around 110 nm, considering the size of the nanotubes which are around 100 nm, this indicates the nanotubes are tightly squeezed together thus appears like an integrated structure. The center of focus appears to be constantly fluctuating as the structures are mobile, the nanotube size seems to be larger in dimension on the Z axis in confocal movie due to its poor axial resolution. Confocal scans (left) are shown for comparison with STED scans (right). The movies are played with a frame rate of 12 fps.

## Data Availability

All study data are included in the article and/or supporting information.

## References

[1] S. Marchi, S. Patergnani, P. Pinton, The endoplasmic reticulum–mitochondria connection: One touch, multiple functions. Biochim. Biophys. Acta **1837**, 461–469 (2014). 24211533 10.1016/j.bbabio.2013.10.015

[2] L. Westrate, J. Lee, W. Prinz, G. Voeltz, Form follows function: The importance of endoplasmic reticulum shape. Ann. Rev. Biochem. **84**, 791–811 (2015). 25580528 10.1146/annurev-biochem-072711-163501

[3] Y. Shibata, G. K. Voeltz, T. A. Rapoport, Rough sheets and smooth tubules. Cell **126**, 435–439 (2006). 16901774 10.1016/j.cell.2006.07.019

[4] G. K. Voeltz, W. A. Prinz, Y. Shibata, J. M. Rist, T. A. Rapoport, A class of membrane proteins shaping the tubular endoplasmic reticulum. Cell **124**, 573–586 (2006). 16469703 10.1016/j.cell.2005.11.047

[5] L. Holmer, H. Worman, Inner nuclear membrane proteins: Functions and targeting. Cellul. Mol. Life Sci. **58**, 1741–1747 (2001). 10.1007/PL00000813 PMC11337314 11766875

[6] R. N. Collins, How the ER stays in shape. Cell **124**, 464–466 (2006). 16469692 10.1016/j.cell.2006.01.017

[7] Y. Shibata , Mechanisms determining the morphology of the peripheral ER. Cell **143**, 774–788 (2010). 21111237 10.1016/j.cell.2010.11.007 PMC3008339

[8] L. K. Schroeder , Dynamic nanoscale morphology of the ER surveyed by STED microscopy. J. Cell Biol. **218**, 83–96 (2019). 30442642 10.1083/jcb.201809107 PMC6314542

[9] Y. G. Zhao, H. Zhang, Phase separation in membrane biology: The interplay between membrane-bound organelles and membraneless condensates. Dev. Cell **55**, 30–44 (2020). 32726575 10.1016/j.devcel.2020.06.033

[10] W. T. Snead , Membrane surfaces regulate assembly of ribonucleoprotein condensates. Nat. Cell Biol. **24**, 461–470 (2022). 35411085 10.1038/s41556-022-00882-3 PMC9035128

[11] W. Ma, C. Mayr, A membraneless organelle associated with the endoplasmic reticulum enables 3′ UTR-mediated protein-protein interactions. Cell **175**, 1492–1506.e19 (2018). 30449617 10.1016/j.cell.2018.10.007 PMC6711188

[12] M. Alenquer , Influenza A virus ribonucleoproteins form liquid organelles at endoplasmic reticulum exit sites. Nat. Commun. **10**, 1629 (2019). 30967547 10.1038/s41467-019-09549-4 PMC6456594

[13] S. Parashar , Endoplasmic reticulum tubules limit the size of misfolded protein condensates. eLife **10**, e71642 (2021). 34467852 10.7554/eLife.71642 PMC8486381

[14] J. R. van Weering, P. J. Cullen, “Membrane-associated cargo recycling by tubule-based endosomal sorting” in Seminars in Cell & Developmental Biology (Elsevier, 2014), pp. 40–47. 10.1016/j.semcdb.2014.03.015 24641888

[15] X. Wang, H.-H. Gerdes, Transfer of mitochondria via tunneling nanotubes rescues apoptotic PC12 cells. Cell Death Differ. **22**, 1181–1191 (2015). 25571977 10.1038/cdd.2014.211 PMC4572865

[16] K. He , Intercellular transportation of quantum dots mediated by membrane nanotubes. Acs Nano **4**, 3015–3022 (2010). 20524630 10.1021/nn1002198

[17] S. Sowinski , Membrane nanotubes physically connect T cells over long distances presenting a novel route for HIV-1 transmission. Nat. Cell Biol. **10**, 211–219 (2008). 18193035 10.1038/ncb1682

[18] M. D. Kolba , Tunneling nanotube-mediated intercellular vesicle and protein transfer in the stroma-provided imatinib resistance in chronic myeloid leukemia cells. Cell Death Dis. **10**, 1–16 (2019). 10.1038/s41419-019-2045-8 PMC6817823 31659149

[19] J. Hurtig, D. T. Chiu, B. Önfelt, Intercellular nanotubes: Insights from imaging studies and beyond. Wiley Interdiscip. Rev. Nanomed. Nanobiotechnol. **2**, 260–276 (2010). 20166114 10.1002/wnan.80 PMC5602582

[20] D. Roy, J. Steinkühler, Z. Zhao, R. Lipowsky, R. Dimova, The mechanical tension of biomembranes can be measured by super resolution (STED) microscopy of force-induced nanotubes. Nano Lett. **20**, 3185–3191 (2020). 32320255 10.1021/acs.nanolett.9b05232 PMC7304919

[21] Z. Zhao , Super-resolution imaging of highly curved membrane structures in giant vesicles encapsulating molecular condensates. Adv. Mater. **34**, 2106633 (2022). 10.1002/adma.202106633 34710248

[22] I. Romero-Brey, R. Bartenschlager, Membranous replication factories induced by plus-strand RNA viruses. Viruses **6**, 2826–2857 (2014). 25054883 10.3390/v6072826 PMC4113795

[23] C. S. Goldsmith , Ultrastructural characterization of SARS coronavirus. Emerg. Infect. Dis. **10**, 320 (2004). 15030705 10.3201/eid1002.030913 PMC3322934

[24] K. H. D. Crawford , Protocol and reagents for pseudotyping lentiviral particles with SARS-CoV-2 spike protein for neutralization assays. Viruses **12**, 513 (2020). 32384820 10.3390/v12050513 PMC7291041

[25] A. M. Syed , Rapid assessment of SARS-CoV-2–evolved variants using virus-like particles. Science **374**, 1626–1632 (2021). 34735219 10.1126/science.abl6184 PMC9005165

[26] M. A. McNiven, H. M. Thompson, Vesicle formation at the plasma membrane and trans-golgi network: The same but different. Science **313**, 1591–1594 (2006). 16973870 10.1126/science.1118133

[27] R. Dimova, C. Marques, The Giant Vesicle Book (Taylor & Francis Group, LLC., Boca Raton, 2019), 10.1201/9781315152516.

[28] R. Dimova, Giant vesicles and their use in assays for assessing membrane phase state, curvature, mechanics, and electrical properties. Annu. Rev. Biophys. **48**, 93–119 (2019). 30811220 10.1146/annurev-biophys-052118-115342

[29] N. Marušič , Constructing artificial respiratory chain in polymer compartments: Insights into the interplay between bo3 oxidase and the membrane. Proc. Natl. Acad. Sci. U.S.A. **117**, 15006–15017 (2020). 32554497 10.1073/pnas.1919306117 PMC7334566

[30] Y. Li, R. Lipowsky, R. Dimova, Membrane nanotubes induced by aqueous phase separation and stabilized by spontaneous curvature. Proc. Natl. Acad. Sci. U.S.A. **108**, 4731–4736 (2011). 21383120 10.1073/pnas.1015892108 PMC3064332

[31] Y. Liu, J. Agudo-Canalejo, A. Grafmüller, R. Dimova, R. Lipowsky, Patterns of flexible nanotubes formed by liquid-ordered and liquid-disordered membranes. ACS Nano **10**, 463–474 (2016). 26588094 10.1021/acsnano.5b05377

[32] R. Dimova, R. Lipowsky, Giant vesicles exposed to aqueous two-phase systems: Membrane wetting, budding processes, and spontaneous tubulation. Adv. Mater. Interfaces **4**, 1600451 (2017).

[33] Y. Wu , Contacts between the endoplasmic reticulum and other membranes in neurons. Proc. Natl. Acad. Sci. U.S.A. **114**, E4859–E4867 (2017). 28559323 10.1073/pnas.1701078114 PMC5474793

[34] L. M. Dominak, C. D. Keating, Polymer encapsulation within giant lipid vesicles. Langmuir **23**, 7148–7154 (2007). 17516666 10.1021/la063687v

[35] S. Sankhagowit , The dynamics of giant unilamellar vesicle oxidation probed by morphological transitions. Biochim. Biophys. Acta **1838**, 2615–2624 (2014). 24998358 10.1016/j.bbamem.2014.06.020

[36] S. Sankhagowit, E. Y. Lee, G. C. Wong, N. Malmstadt, Oxidation of membrane curvature-regulating phosphatidylethanolamine lipid results in formation of bilayer and cubic structures. Langmuir **32**, 2450–2457 (2016). 26866900 10.1021/acs.langmuir.5b04332 PMC6559366

[37] I. O. Bacellar , Permeability of DOPC bilayers under photoinduced oxidation: Sensitivity to photosensitizer. Biochim. Biophys. Acta **1860**, 2366–2373 (2018). 10.1016/j.bbamem.2018.06.001 29886032

[38] A. H. Bahrami, G. Hummer, Formation and stability of lipid membrane nanotubes. ACS Nano **11**, 9558–9565 (2017). 28873296 10.1021/acsnano.7b05542

[39] L. M. Dominak, C. D. Keating, Macromolecular crowding improves polymer encapsulation within giant lipid vesicles. Langmuir **24**, 13565–13571 (2008). 18980360 10.1021/la8028403

[40] L. M. Dominak, D. M. Omiatek, E. L. Gundermann, M. L. Heien, C. D. Keating, Polymeric crowding agents improve passive biomacromolecule encapsulation in lipid vesicles. Langmuir **26**, 13195–13200 (2010). 20695558 10.1021/la101903r PMC2919175

[41] J. R. Friedman , ER tubules mark sites of mitochondrial division. Science **334**, 358–362 (2011). 21885730 10.1126/science.1207385 PMC3366560

[42] X. Liu , Mechanical force induces DRP1-dependent asymmetrical mitochondrial fission for quality control. bioRxiv [Preprint] (2022). 10.1101/2022.10.27.513965 (Accessed 9 November 2023).

[43] H. Wang, F. M. Kelley, D. Milovanovic, B. S. Schuster, Z. Shi, Surface tension and viscosity of protein condensates quantified by micropipette aspiration. Biophys. Rep. **1**, 100011 (2021). 10.1016/j.bpr.2021.100011 PMC9563586 36247368

[44] C. Kilchert, J. Weidner, C. Prescianotto-Baschong, A. Spang, Defects in the secretory pathway and high Ca2+ induce multiple P-bodies Mol. Biol. Cell **21**, 2624–2638 (2010). 20519435 10.1091/mbc.E10-02-0099 PMC2912349

[45] J. E. Lee, P. I. Cathey, H. Wu, R. Parker, G. K. Voeltz, Endoplasmic reticulum contact sites regulate the dynamics of membraneless organelles. Science **367**, eaay7108 (2020). 32001628 10.1126/science.aay7108 PMC10088059

[46] J. H. Hurley, L. N. Young, Mechanisms of autophagy initiation. Annu. Rev. Biochem. **86**, 225–244 (2017). 28301741 10.1146/annurev-biochem-061516-044820 PMC5604869

[47] N. Wang, Y. Shibata, J. A. Paulo, S. P. Gygi, T. A. Rapoport, A conserved membrane curvature-generating protein is crucial for autophagosome formation in fission yeast. Nat. Commun. **14**, 4765 (2023). 37553386 10.1038/s41467-023-40530-4 PMC10409813

[48] Z. Xie, D. J. Klionsky, Autophagosome formation: Core machinery and adaptations. Nat. Cell Biol. **9**, 1102–1109 (2007). 17909521 10.1038/ncb1007-1102

[49] T. J. Melia, A. H. Lystad, A. Simonsen, Autophagosome biogenesis: From membrane growth to closure. J. Cell Biol. **219**, e202002085 (2020). 32357219 10.1083/jcb.202002085 PMC7265318

[50] H. Nakatogawa, Mechanisms governing autophagosome biogenesis. Nat. Rev. Mol. Cell Biol. **21**, 439–458 (2020). 32372019 10.1038/s41580-020-0241-0

[51] Y. Fujioka , Phase separation organizes the site of autophagosome formation. Nature **578**, 301–305 (2020). 32025038 10.1038/s41586-020-1977-6

[52] Y. Fujioka, N. N. Noda, Biomolecular condensates in autophagy regulation. Curr. Opin. Cell Biol. **69**, 23–29 (2021). 33445149 10.1016/j.ceb.2020.12.011

[53] X. Ma, P. Li, L. Ge, Targeting of biomolecular condensates to the autophagy pathway. Trends Cell Biol. **33**, 505–516 (2023). 36150962 10.1016/j.tcb.2022.08.006

[54] A. Mangiarotti, N. Chen, Z. Zhao, R. Lipowsky, R. Dimova, Wetting and complex remodeling of membranes by biomolecular condensates. Nat. Commun. **14**, 2809 (2023). 37217523 10.1038/s41467-023-37955-2 PMC10203268

[55] A. Mangiarotti , Biomolecular condensates modulate membrane lipid packing and hydration. Nat. Commun. **14**, 6081 (2023). 37770422 10.1038/s41467-023-41709-5 PMC10539446

[56] T. Lu , Endocytosis of coacervates into liposomes. J. Am. Chem. Soc. **144**, 13451–13455 (2022). 35878395 10.1021/jacs.2c04096 PMC9354246

[57] A. Mangiarotti , Photoswitchable endocytosis of biomolecular condensates in giant vesicles. Adv. Sci. (2024), 10.1101/2024.01.10.574984. PMC11187966 38582523

[58] A. Mangiarotti, R. Dimova, Biomolecular condensates in contact with membranes. Annu. Rev. Biophys., 10.1146/annurev-biophys-030722-121518 (2024). 38360555

[59] Y. Liu, R. Lipowsky, R. Dimova, Concentration dependence of the interfacial tension for aqueous two-phase polymer solutions of dextran and polyethylene glycol. Langmuir **28**, 3831–3839 (2012). 22292882 10.1021/la204757z

[60] Z. Zhao , Molar mass fractionation in aqueous two-phase polymer solutions of dextran and poly (ethylene glycol). J. Chromatograp. **1452**, 107–115 (2016). 10.1016/j.chroma.2016.04.075 27155914

[61] Y. Li, R. Lipowsky, R. Dimova, Transition from complete to partial wetting within membrane compartments. J. Am. Chem. Soc. **130**, 12252–12253 (2008). 18712871 10.1021/ja8048496

[62] R. S. Gracià, N. Bezlyepkina, R. L. Knorr, R. Lipowsky, R. Dimova, Effect of cholesterol on the rigidity of saturated and unsaturated membranes: Fluctuation and electrodeformation analysis of giant vesicles. Soft Matter **6**, 1472–1482 (2010).

[63] H. A. Faizi, C. J. Reeves, V. N. Georgiev, P. M. Vlahovska, R. Dimova, Fluctuation spectroscopy of giant unilamellar vesicles using confocal and phase contrast microscopy. Soft Matter **16**, 8996–9001 (2020). 10.1039/d0sm00943a 32966528

[64] M. Heinrich, A. Tian, C. Esposito, T. Baumgart, Dynamic sorting of lipids and proteins in membrane tubes with a moving phase boundary. Proc. Natl. Acad. Sci. U.S.A. **107**, 7208–7213 (2010). 20368457 10.1073/pnas.0913997107 PMC2867702

[65] R. Lipowsky, Response of membranes and vesicles to capillary forces arising from aqueous two-phase systems and water-in-water droplets. The J. Phys. Chem. B **122**, 3572–3586 (2018). 29465241 10.1021/acs.jpcb.7b10783

[66] H. Kusumaatmaja, Y. Li, R. Dimova, R. Lipowsky, Intrinsic contact angle of aqueous phases at membranes and vesicles. Phys. Rev. Lett. **103**, 238103 (2009). 20366179 10.1103/PhysRevLett.103.238103

