# Supplementary material for: Membrane nanotubes transform into double-membrane sheets at condensate droplets
Source: Proc Natl Acad Sci U S A. 2024 Jun 20;121(26):e2321579121. doi: 10.1073/pnas.2321579121 (PMC11214096; doi:10.1073/pnas.2321579121)
Supplement: Supplementary file 1 — Appendix 01 (PDF) [file pnas.2321579121.sapp.pdf]

# Supporting Information

## Membrane nanotubes transform into double-membrane sheets at condensate droplets

Ziliang Zhao[1,2,3]*, Vahid Satarifard[1,4], Reinhard Lipowsky[1] and Rumiana Dimova[1]*

[1] *Max Planck Institute of Colloids and Interfaces, Science Park Golm, 14476 Potsdam, Germany*
[2] *Leibniz Institute of Photonic Technology e.V., Albert-Einstein-Straße 9, 07745 Jena, Germany*
[3] *Institute of Applied Optics and Biophysics, Friedrich-Schiller-University Jena, Max-Wien Platz 1, 07743 Jena, Germany*
[4] *Yale Institute for Network Science, Yale University, New Haven, CT 06520, USA*

* *Address for correspondence: Ziliang.Zhao@leibniz-ipht.de; Rumiana.Dimova@mpikg.mpg.de*

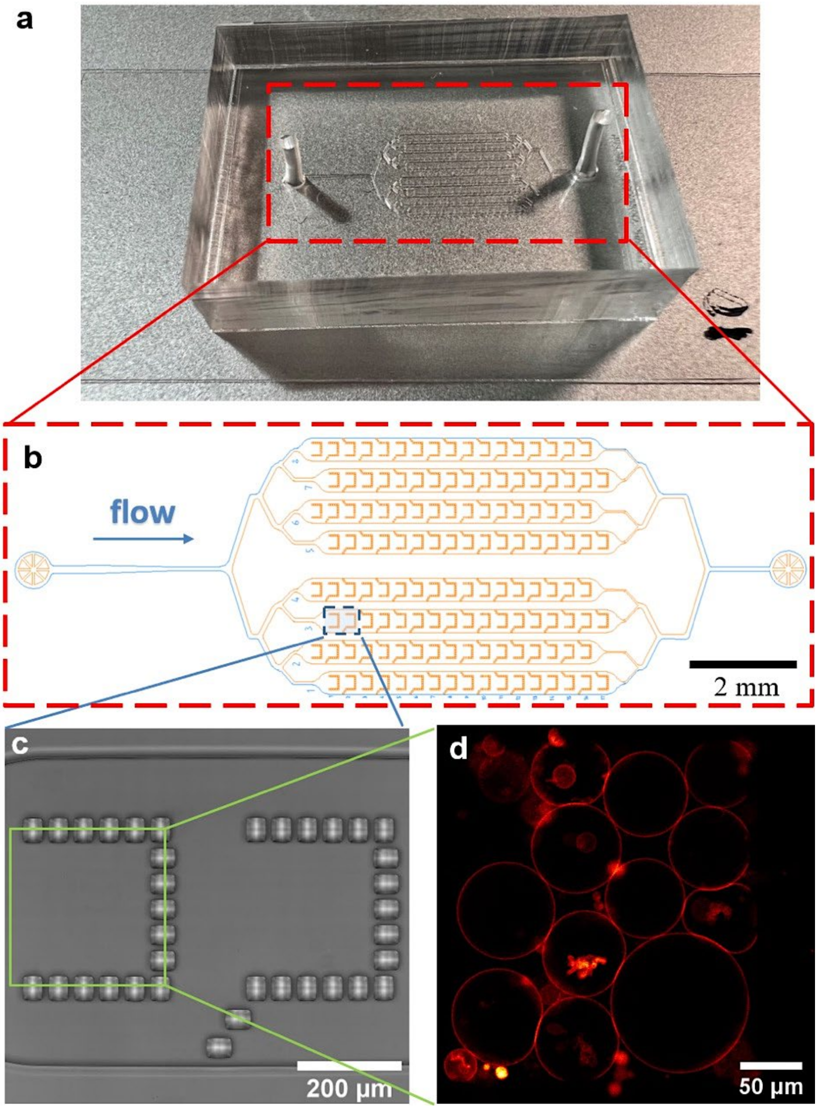


**Figure S1. GUV trapping in a microfluidic chip. (a)** A picture of PDMS-based microfluidic chip (roughly 2 cm × 3 cm × 0.8 cm) used for GUV trapping and fluid exchange. **(b**) Sketch of microfluidic chip structures as indicated in the red dashed rectangular region in (a); the chip contains 8 flow channels, each equipped with 17 GUV traps. **(c)** A bright field image of the two traps indicated by the blue rectangular area in (b). **(d)** Confocal microscopy cross section of the GUVs collected in the trap indicated in the green area in (c).

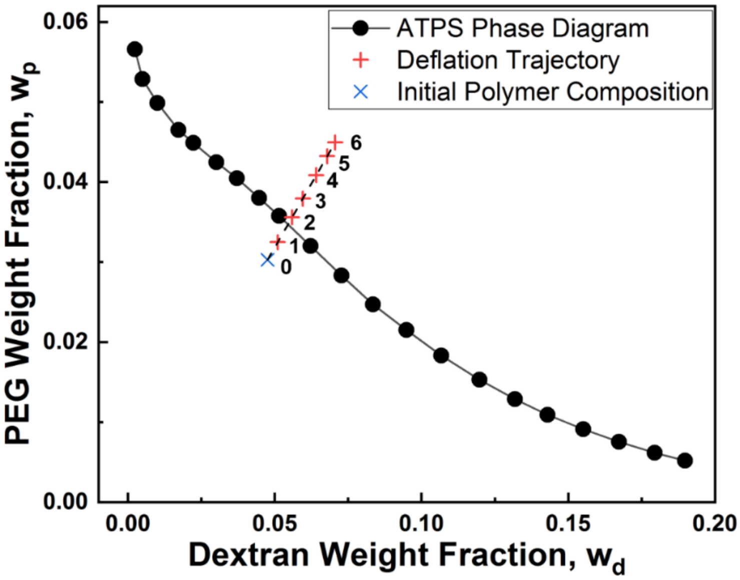


**Figure S2. Osmotic deflation trajectory in the PEG/dextran system.** High precision phase diagram of aqueous dextran and PEG solutions is displayed in black circles and dashed deflation trajectory with blue and red crosses. Point 0 indicates the initial polymer weight concentration of ($w_d$, $w_p$) = (0.0476, 0.0303). The GUVs were deflated in a step-wise manner by a hypertonic solution (dextran/PEG solution of D/P=1, i.e. 3.54%, 3.54% weight fraction with increasing sucrose concentrations) which leads to interior compositions 1, 2, 3, 4, 5, and 6 corresponding to osmolarity ratio r=1.2, 1.4, 1.6, 1.8, 2.0 and 2.2, respectively.

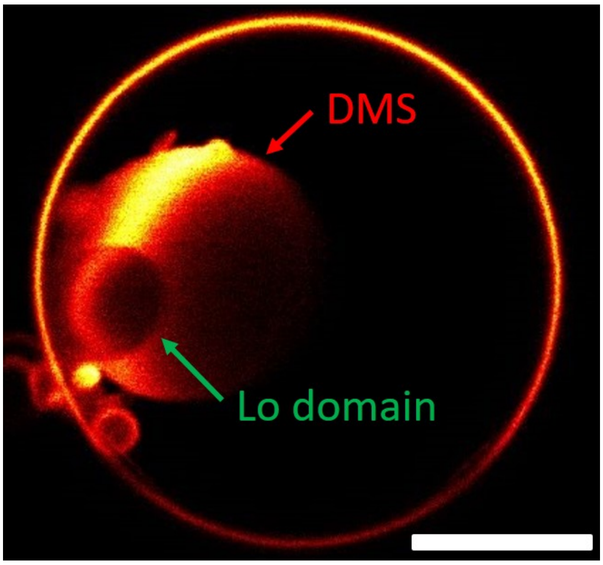


**Figure S3. Confocal microscopy image of a double membrane sheet (DMS) in a GUV exhibiting liquid-disordered and liquid-ordered Ld/Lo (DOPC:DPPC:cholesterol 35:35:30) phase coexistence.** Both phases are observed on the DMS (red arrow), with Lo domain (green arrow) depleted of fluorescence signal. Single component GUVs with DOPC were also probed showing DMS appearances as for the liquid-disordered GUVs in the main text. Scale bar: 10 μm.

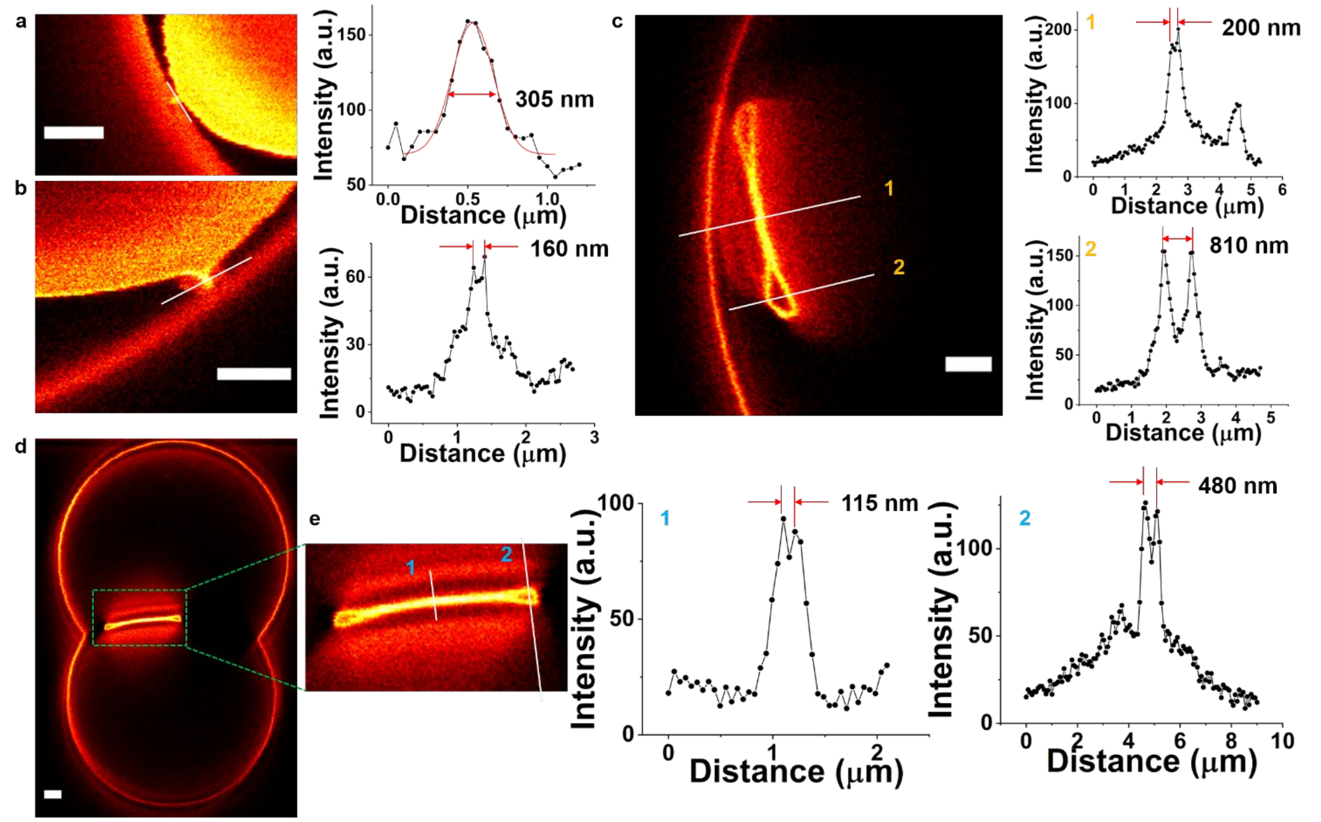


**Figure S4. 2D & 3D STED line profile analysis on membrane neck and DMS. (a)** 2D STED xy-scan with a pixel size of 50 nm and corresponding line profile on the right (same GUV as in Fig. 2a). The red solid line represents optimal Gaussian fit curve, which yields for the membrane neck thickness of 305 nm (from the full width at half maximum, FWHM). If membrane necks are imaged in a larger scanning region as here, they can appear thicker because of lateral and out-of-focus movement during scanning. **(b)** 2D STED xy-scan with a pixel size of 40 nm (same GUV as in. Fig. 2b): imaging was performed in a much smaller region than in panel (a) so that adequate membrane neck detail can be revealed by shorter STED acquisition time to avoid artefacts due to the displacement of the DMS. The corresponding numbered line profile is shown on the right. The peak-to-peak distance indicates the distance between the lines crossing the walls of the tubular neck with thickness of 160 nm. **(c)** 3D STED xy-scan with a pixel size of 50 nm and the corresponding DMS line profiles (indicated with 1 and 2 on the image) shown on the right. The peak-to-peak distance indicates the DMS has a middle thickness of 200 nm and rim thickness of 810 nm. **(d, e)** 3D STED xz-scan with a pixel size of 80 nm on a Janus GUV (same as in Fig. 2d) and the enlarged image of the DMS. The corresponding numbered line profiles are shown on the right, indicating a middle thickness of 115 nm and a rim thickness of 480 nm for the DMS. Scale bars: 2 μm.

## Membrane Nanotubes and Double-Membrane Sheets (DMSs) Adhering to a Liquid-Liquid Interface.

**Liquid-liquid phase separation and partial wetting.** For the GUVs studied here, the interior solution undergoes phase separation into the two liquid phases $\alpha$ and $\beta$, separated by an $\alpha\beta$ interface (in-wetting). The liquid phase of the exterior solution is denoted by $\gamma$. The $\alpha\beta$ interface has the shape of a spherical cap with curvature radius $R_{\alpha\beta}$ as follows from the Laplace equation. When one of the two phases $\alpha$ or $\beta$ completely wets the GUV membrane, the $\alpha\beta$ interface forms a complete sphere. When the two phases partially wet the GUV membrane, the $\alpha\beta$ interface meets this membrane along its global contact line. The global contact line leads to an apparent kink of the GUV shape which is directly visible in the optical image of this shape. Furthermore, for partial wetting, any membrane protrusion filled with $\gamma$ phase adheres to the $\alpha\beta$ interface because the adhesion reduces the energy of the protrusion. When the

protrusion comes into contact with the $\alpha\beta$ interface, this interface forms additional, local contact lines with the membrane of the protrusion.

**Total energy of membrane protrusion.** The energy of any membrane protrusion that adheres to a liquid-liquid ($\alpha\beta$) interface consists of two contributions, arising from the membrane's curvature elasticity and from its adhesion to the interface. The membrane protrusion can be a nanotube or a double-membrane sheet (DMS) as specified further below.

**Bending energy of membrane protrusion.** In the experiments, the membrane protrusions undergo shape transformations but do not change their topology, which implies that the curvature energy of the membrane is equal to its bending energy. Using the spontaneous curvature model, the bending energy of the membrane protrusion is given by

$$E_{be} = 2\kappa \int dA\ 2\kappa(M - m)^2 \qquad (S1)$$

which represents the integral over the total area $A$ of the protrusion. This bending energy is proportional to the bending rigidity $\kappa$ and depends on the deviation of the (local) mean curvature $M$ of the membrane from its spontaneous curvature $m$.

**Adhesion energy of membrane protrusion.** The adhesion energy of the membrane protrusion is provided by the reversible work that one has to expend in order to remove the protrusion from the interface and to immerse it into the PEG-rich $\alpha$ phase. Alternatively, we could also move the membrane segment into the dextran-rich $\beta$ phase. The two adhesion energies differ by the reversible work to move the membrane segment from full immersion in the $\alpha$ phase to full immersion in the $\beta$ phase. When the membrane protrusion adheres to the $\alpha\beta$ interface and forms a finite contact angle with this interface, corresponding to partial wetting of the membrane by both the $\alpha$ and the $\beta$ phase, the membrane protrusion is divided up into two segments, the $\alpha\gamma$ segment between the $\alpha$ and the $\gamma$ phase as well as the $\beta\gamma$ segment between the $\beta$ and the $\gamma$ phase. Both segments meet the $\alpha\beta$ interface along the local contact lines. Likewise, the total area $A$ of the membrane protrusion is divided up into two contact areas according to

$$A = A_{\alpha\gamma} + A_{\beta\gamma} \qquad (S2)$$

The adhesion energy of the membrane protrusion then has the form

$$E_{ad} = \Sigma_{\alpha\gamma}A_{\alpha\gamma} + \Sigma_{\beta\gamma}A_{\beta\gamma} + \Sigma_{\alpha\beta}\left(A_{\alpha\beta} - \Delta A_{\alpha\beta}\right) - \left[\Sigma_{\alpha\gamma}\left(A_{\alpha\gamma} + A_{\beta\gamma}\right) + \Sigma_{\alpha\beta}A_{\alpha\beta}\right] \qquad (S3)$$

where $\Delta A_{\alpha\beta}$ is the reduction of the interfacial area $A_{\alpha\beta}$ by the adhering protrusion. The right-hand side of this equation contains several terms that cancel out which leads to the simpler expression

$$E_{ad} = (\Sigma_{\beta\gamma} - \Sigma_{\alpha\gamma})A_{\beta\gamma} - \Sigma_{\alpha\beta}\Delta A_{\alpha\beta} \qquad (S4)$$

In equilibrium, the intrinsic contact angle $\theta_{in}$ at the contact line satisfies the force balance relation

$$\Sigma_{\beta\gamma} - \Sigma_{\alpha\gamma} = \Sigma_{\alpha\beta}\cos\theta_{in} \qquad (S5)$$

where contributions arising from the line tension of the contact line have been ignored. The relation in Eq. (S5) looks like the classical Young equation for contact angles at a rigid surface but is, in fact, a consequence of the requirement that the membrane has no kinks on nanoscopic scales. Using Eq. (S5) to eliminate $\Sigma_{\beta\gamma} - \Sigma_{\alpha\gamma}$ from Eq. (S4) the adhesion energy attains the compact form

$$E_{ad} = \Sigma_{\alpha\beta}\left(\cos\theta_{in}\, A_{\beta\gamma} - \Delta A_{\alpha\beta}\right) \qquad (S6)$$

This adhesion energy is proportional to the interfacial tension $\Sigma_{\alpha\beta}$ and depends on the intrinsic contact angle $\theta_{in}$ as well as on the segment areas $A_{\beta\gamma}$ and $\Delta A_{\alpha\beta}$.

**Membrane nanotube adhering to $\alpha\beta$ interface.** We consider a single nanotube with area $A_{nt}$. The adhesion between this nanotube and the $\alpha\beta$ interface leads to a local contact line that consists of two circular segments. These two circular contact line segments have the same radius, which is close to $R_{\alpha\beta}$ but in general different from $R_{\alpha\beta}$. The two circular contact line segments partition the area $A_{nt}$ of the nanotube into two toroidal segments $\alpha\gamma$ and $\beta\gamma$, which are in contact with the $\alpha$ and $\beta$ phases, respectively. Thus, we can decompose the tube area $A_{nt}$ according to

$$A_{nt} = A_{\alpha\gamma} + A_{\beta\gamma} \tag{S7}$$

where $A_{\alpha\gamma}$ and $A_{\beta\gamma}$ represent the membrane area of the $\alpha\gamma$ and $\beta\gamma$ segments, respectively. These geometric features apply to any cross-section of the nanotube.

For computational simplicity, the nanotube is now taken to have a circular cross-section of radius $R_{nt}$. If we first ignore the confinement of the $\alpha\beta$ interface by the mother vesicle, the torus represents a surface of revolution and can be constructed by revolving the circular cross-section around an axis through the center of the spherical $\alpha\beta$ cap. The center of the circular cross-section then runs through another circle with radius $R_{la}$ and circumference $2\pi R_{la}$. The area of such a complete torus is $2\pi R_{nt} \times 2\pi R_{la}$. Because the extension of the $\alpha\beta$ interface is confined by the mother vesicle, the center of the circular cross-section runs only through a circular segment of length $L < 2\pi R_{la}$. The area of the nanotube is then given by

$$A_{nt} = A_{\alpha\gamma} + A_{\beta\gamma} = 2\pi R_{nt} L \tag{S8}$$

Another area which is crucial in order to compute the adhesion energy of the tube, is the area $\Delta A_{\alpha\beta}$ by which the interfacial area $A_{\alpha\beta}$ is reduced when the nanotube adheres to the $\alpha\beta$ interface. Thus, in the presence of the adhering nanotube, the interfacial area is reduced from $A_{\alpha\beta}$ to $A_{\alpha\beta} - \Delta A_{\alpha\beta}$.

**Areas expressed in terms of angles.** The areas $A_{\alpha\gamma}$, $A_{\beta\gamma}$, and $\Delta A_{\alpha\beta}$ are all proportional to the tube area $A_{nt}$ with proportionality factors that depend on the intrinsic contact angle $\theta_{in}$ and on another angle $\phi$ as defined in Fig. S5a. Using some trigonometric relations, this angle can be shown to satisfy the relationship

$$\phi = \operatorname{arccot}\left[\frac{R_{\alpha\beta}}{R_{nt}\sin\theta_{in}} + \cot\theta_{in}\right] \tag{S9}$$

For large $x$, the inverse cotangens function behaves as $\operatorname{arccot} x \approx 1/x$, which implies

$$\phi \approx \frac{\epsilon}{\sin\theta_{in}} \text{ for small } \epsilon = R_{nt}/R_{\alpha\beta} \tag{S10}$$

Using this asymptotic behavior of $\phi$, we can also obtain the leading corrections of all $\phi$-dependent quantities for small $\epsilon$.

Furthermore, trigonometric considerations also lead the segment areas

$$A_{\alpha\gamma} = \frac{(\pi - \theta_{in} + \phi)A_{nt}}{\pi} \tag{S11}$$

$$A_{\beta\gamma} = \frac{(\theta_{in} - \phi)A_{nt}}{\pi} \tag{S12}$$

and

$$\Delta A_{\alpha\beta} = \frac{\phi(\cot\psi\sin\theta_{in} - \cos\theta_{in})A_{nt}}{\pi} \tag{S13}$$

**Total energy of adhering nanotube.** The total energy of the nanotube consists of the bending energy of its membrane and the adhesion energies of the membrane with the $\alpha$ and $\beta$ phases.

**Bending energy of nanotube.** The bending energy of the tube is given by Eq. (S1) where the integral runs over the area $A_{nt}$ of the nanotube. If the shape of the nanotube has a constant mean curvature, $M_1$, the bending energy would become

$$E_{be}^{nt} = \sum_{j=\alpha,\beta} \left[2\kappa\left(M_1 - m_{j\gamma}\right)^2 A_{j\gamma}\right] \tag{S14}$$

We can express the interfacial areas $A_{\alpha\gamma}$ and $A_{\beta\gamma}$ in terms of the total interfacial nanotube areas using the trigonometric relations: $A_{\alpha\gamma} = [(\pi - \theta_{in} + \phi)/\pi]\, A_{nt}$ and $A_{\beta\gamma} = [(\theta_{in} - \phi)/\pi]\, A_{nt}$, see Figure S5a. The expression in Eq. (S14) applies to a cylindrical tube but not to the toroidal tube defined above because a torus with circular cross-section does not have a constant mean curvature. It is not difficult to see that the mean curvature *M* of the toroidal torus varies within the range $M_{low} \leq M \leq M_{upp}$, with the lower bound $M_{low} = (1/2)[(1/R_{nt}) + 1/(R_{la} + R_{nt})]$ and the upper bound $M_{upp} = (1/2)[(1/R_{nt}) + 1/(R_{la} - R_{nt})]$. We now define the mean curvature

$$M_o \equiv \frac{1}{2}\left[\frac{1}{R_{nt}} + \frac{1}{R_{la}}\right] \tag{S15}$$

which satisfies $M_{low} \leq M_o \leq M_{upp}$, and represents the mean curvature of those tube segments that have the same distance from the rotational axis as the center of the circular cross-section. In the experiments, the ratio $\epsilon \equiv R_{nt}/R_{la}$ is small and of the order of $10^{-3}$ ($R_{nt}$=46.5 nm, and $R_{la}$=24 µm for the DMS in Fig. 2d). We express the deviations of the mean curvature from its lower and upper bound values in terms of $\epsilon$. The lower and upper bound then behave as $M_{low} \approx M_o - \epsilon^2/2R_{nt}$ and the upper bound $M_{upp} \approx M_o + \epsilon^2/2R_{nt}$, for small $\epsilon$. As a consequence, the local mean curvature *M* behaves as $M \approx M_o = 0.011\ [1/nm]$ with small corrections of the order of $10^{-6}$.

**Adhesion energy of nanotube.** The adhesion energy of the nanotube is provided by Eq. (S6) which depends on two material parameters, the interfacial tension $\Sigma_{\alpha\beta}$ of the $\alpha\beta$ interface and the intrinsic contact angle $\theta_{in}$ as well as on the two geometric quantities, the membrane areas $A_{\beta\gamma}$ and $\Delta A_{\alpha\beta}$. The area $A_{\beta\gamma}$ represents the membrane area of the tube segment in contact with the $\beta$ phase and the area $\Delta A_{\alpha\beta}$ represents the reduction of the interfacial area by the adhering nanotube. For the adhering nanotube, the membrane areas $A_{\beta\gamma}$ and $\Delta A_{\alpha\beta}$ are provided by the expressions in Eqs. (S11)-(S13). When we insert these expressions into Eq. (S6) we obtain the adhesion energy in the compact form

$$E_{ad}^{nt} = \Sigma_{\alpha\beta} A_{nt} \frac{(\theta_{in}\cos\theta_{in} - \phi\cot\phi\sin\theta_{in})}{\pi} \tag{S16}$$

**Double-membrane sheet (DMS) adhering to $\alpha\beta$ interface**

**Geometry of adhering DMS.** The experimental observations of the DMSs show that they are axisymmetric, with a rotational symmetry axis that is perpendicular to the $\alpha\beta$ interface. In addition, the STED images provide detailed cross-sections of the DMSs and of the associated contours of the double-membranes. We now consider one such DMS and its shape contour for a normal section that contains the rotational symmetry axis. The experimentally observed contour consists of three segments: (i) two long circular segments that are parallel to each other and to the contour of the $\alpha\beta$ interface. These two circular segments correspond to two spherical membrane caps, which are separated by 115 nm from each other (Fig. 2e). The two spherical cap segments are connected by another membrane segment that resembles part of a distorted toroidal segment. The cross section of this latter segment has a diameter of 480 nm (Fig. 2e).

**Shape contour fitted with five circular segments.** The experimentally observed shape contour is fitted using five circular segments, labeled by $i = 1, 2, 3, 4$ and 5. Here and below, we take these labels to be: $i = 1$ for the spherical cap segment in contact with the dextran-rich β phase; $i = 5$ for the spherical cap segment in contact with the PEG-rich α phase; and $i = 2, 3$, and 4 for the three circular segments that are fitted to the contour of the toroidal segment at the rim of the sheet, see Fig. S5b,c. The axis of rotational symmetry is taken to be the $z$-axis. This symmetry axis intersects the DMS at two points, $z = z_{np}$ and $z = z_{sp}$, corresponding to the north and the south pole of the DMS. We take the north pole to be located above the south pole, which implies $z_{np} > z_{sp}$.

**Parametrization of shape contour.** We parametrize the shape contour in terms of its arc length $s$. Each value of $s$ determines a unique point on the contour. The south pole is located at $s = 0$, the north pole at $s = s_{tot}$ where $s_{tot}$ represents the total arc length of the contour. The shape of the contour is then described by the radial coordinate $r = r(s)$ and the tilt angle $\psi = \psi(s)$, which both vary with the arc length $s$, see Fig. S5c. The radial coordinate $r$ measures the distance of the contour point at $s$ from the $z$-axis, the tilt angle $\psi$ is defined by the normal vector of the shape contour at the contour point $s$. This tilt angle is equal to the angle between the normal vector of the shape contour and the $z$-axis. At the south pole with $r = 0$, the normal vector is parallel to the $z$-axis and the tilt angle $\psi(s = 0) = 0$, see Fig. S5c. The latter is because the normal vector of the shape contour at the contour point $s = 0$ in pointing inward. As we move away from the south pole, the tilt angle may become positive or negative, depending on whether the south pole represents a maximum or a minimum of the shape contour. In Fig. S5c, the shape contour has a maximum at the south pole which implies that the tilt angle becomes positive for small $s$ corresponding to a positive contour curvature $C_1 = d\psi/ds > 0$.

**Principal and mean curvatures of axisymmetric shapes.** The two principal curvatures, $C_1$ and $C_2$, of the axisymmetric shape are given by simple expressions in terms of $\psi$ and $r$. The principal curvature $C_1$ parallel to the contour, which represents the contour curvature, is simply given by

$$C_1 = \frac{d\psi(s)}{ds} \tag{S17}$$

whereas the principal curvature $C_2$ perpendicular to the contour has the form

$$C_2 = \frac{\sin\psi(s)}{r(s)} \tag{S18}$$

and the local mean curvatures of each segment is then given by

$$M_i(s) = \frac{1}{2}\left(\frac{d\psi(s)}{ds} + \frac{\sin\psi(s)}{r(s)}\right) \tag{S19}$$

Each contour segment in Fig. S5c was chosen to be a circular segment. The geometry of the circular segment $S_i$ involves the curvature radius $R_i$ of this segment, $L_i$ the distance of the center of circular segment from the $z$-axis, the opening angle $\omega_i$, and the initial tilt angle $\psi_i$. The total arc length of the circular segment is equal to $R_i\omega_i$. For each circular segment $S_i$, the four geometric parameters $R_i$, $L_i$, $\omega_i$, and $\psi_i$ provide four fit parameters. The numerical values of these fit parameters are tabulated in Table S1.

**Total energy of adhering DMS**

**Bending energy of DMS.** The bending energy of the DMS is given by

$$E_{be}^{sh} = \sum_{j=\alpha,\beta}\left[2\kappa\sum_{i=1}^{5}\int ds_i\, 2\pi r(s)\big(M_i(s) - m_{j\gamma}\big)^2\right] \tag{S20}$$

where mean curvatures $M_i(s)$ are defined in Eq. (S19) and $m_{j\gamma}$ is the spontaneous curvature of $\alpha\gamma$ or $\beta\gamma$ segments of the membrane. We calculated the integral in Eq. (S20) using Trapezoidal rule.

**Areas of five membrane segments.** Next, we should calculate the membrane areas $A_i$ of the five membrane segments, which determine the contact areas $A_{\alpha\gamma}$ and $A_{\beta\gamma}$ of the DMS with the $\alpha$ and $\beta$ phase and, thus, the adhesion energy of the DMS. The area for each circular segment is calculated by

$$A_i = 2\pi \int ds_i \, r(s) \qquad (S21)$$

We calculated the integral in Eq. (S21) using Trapezoidal rule. The numerical values of the area segments are tabulated in Table S1.

**Adhesion energy of DMS.** The adhesion energy of the double-membrane sheet is given by

$$E_{ad}^{sh} = \Sigma_{\alpha\beta}\left(\cos\theta_{in} A_{\beta\gamma} - \Delta A_{\alpha\beta}\right) \qquad (S22)$$

as in Eq. (S6) with $A_{\beta\gamma} = XA_3 + A_1 + A_2$, where $X = 0.409$ denotes the fraction of the membrane segment with $i = 3$ that is in contact with the $\beta$ phase.

The morphological transformation from nanotube to DMS requires that the total energy of the energy of DMS wetting the $\alpha\beta$ interface is less than the energy of the membrane nanotubes $E_{ad}^{sh} + E_{be}^{sh} < E_{ad}^{nt} + E_{be}^{nt}$, and at a transition line the energies of interfacial membrane nanotubes and sheets are equal $E_{tot}^{sh} = E_{tot}^{nt}$. Using Eqs. (S14), (S16), (S20) and (S22) and some algebra leads to the critical interfacial tension $\Sigma_{\alpha\beta}^*$ condition

$$\Sigma_{\alpha\beta}^* \equiv \frac{\pi}{A_{pr}}\left[\frac{E_{be}^{sh} - E_{be}^{nt}}{\left(\theta_{\text{in}} - \pi f_{\beta\gamma}\right)\cos\theta_{in} - \phi\cot\phi\sin\theta_{in} + \pi f_{\alpha\beta}}\right] \qquad (S23)$$

where $A_{pr} = A_{nt} = A_{sh}$ a and the two area fractions are defined as $f_{\beta\gamma} = A_{\beta\gamma}/A_{pr}$ and $f_{\alpha\beta} = \Delta A_{\alpha\beta}/A_{pr}$. All the parameters used to calculate the critical interfacial tension are tabulated in Table S1 and S2.

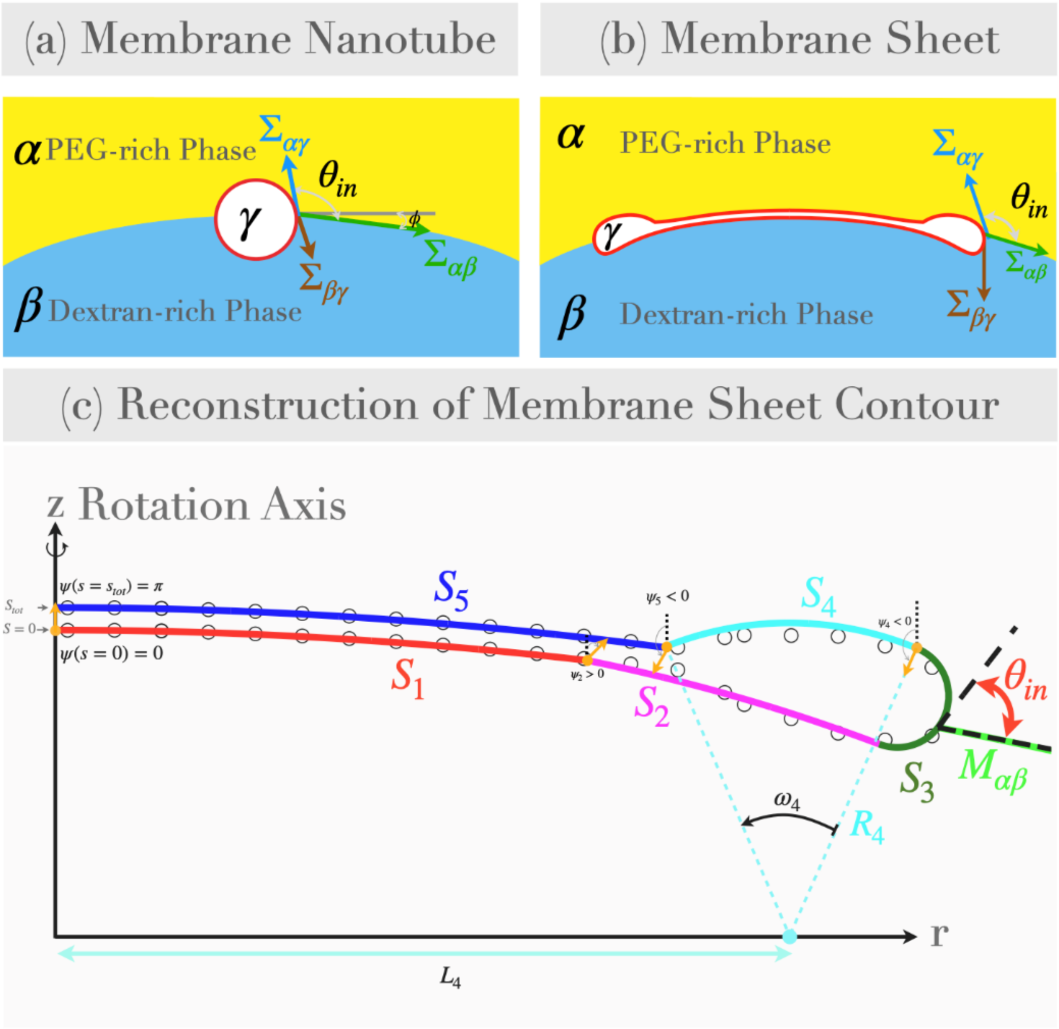


**Figure S5.** The schematic of membrane nanotube **(a)** and DMS **(b)** partially wetted by liquid phases at the $\alpha\beta$ interfaces, namely PEG-rich and dextran-rich phases labeled by $\alpha$ (yellow) and $\beta$ (blue) phases, respectively. The exterior phase is named the $\gamma$ phase (white). Different force balance components and the angle components are shown for both membrane nanotube and DMS. The membrane surface area is illustrated in red. **(c)** Reconstructed DMS contour, and parameters describing the contour in each segment. For segment *S*4 the parameters are defined explicitly. The orange vector shows the normal vector of the shape contour in different parts of the contour, and the tilt angle $\psi_i$ is defined as the angle between normal vector of the shape contour and the *z*-axis.

| Segment *i* | $R_i$ [μm] | $L_i$ [μm] | $\psi_i$ | $\omega_i$ | $C_{1,i}$ [1/μm] | $C_{2,i}$ [1/μm] | $A_i$ [μm$^2$] |
|---|---|---|---|---|---|---|---|
| 1 | 24 | 0.0 | 0.0 | 0.1138 | 0.042 | 0.042 | 23.43 |
| 2 | 9.56 | 0.85 | 0.1976 | 0.1628 | 0.105 | [0.072, 0.084] | 18.33 |
| 3 | 0.26 | 4.31 | 0.3604 | 3.1074 | -3.801 | [-0.219, 0.084] | 22.15 |
| 4 | 1.70 | 3.76 | -2.7471 | 0.7803 | -0.587 | [-0.087, 0.121] | 31.38 |
| 5 | 24 | 0.0 | -3.0113 | 0.1303 | -0.042 | -0.042 | 30.69 |

**Table S1. Parameters of the model.** Fit parameters that are calculated from the circular fit to line profile obtained from the peak-to-peak distance data of STED images and curvature of the liquid-liquid interface. Total DMS area is $A_{sh}$ = 142.51 μm$^2$.

| Parameter | Value | unit |
|---|---|---|
| $m_{j\gamma}$ | [-0.005, 0] | [1/nm] |
| $D_{nt}$ | 93 | [nm] |
| $M_{\alpha\beta}$ | 1/24 | [1/µm] |
| $\kappa$ | 14.9 | [$k_BT$] |
| $\theta_{in}$ | 63 | Deg |
| $f_{\beta\gamma}$ | 0.469 | |
| $f_{\alpha\beta}$ | 0.559 | |

**Table S2. Parameters from Experimental Measurements**. These parameters are directly obtained from experiments. However, the value of the spontaneous curvature is chosen to be the free parameter to calculate the critical interfacial tension and subsequently build the morphology diagram.

## Coexistence of stable/metastable membrane nanotubes and DMSs

In the experiments, we observed that membrane nanotubes and DMSs can coexist. This indicates that nanotubes and DMSs can be stable and/or metastable in part of the phase diagram. To demonstrate this hypothesis, we use a simplified model of DMS with two spherical caps of constant mean curvature ($M = 1/24$ µm$^{-1}$) separated by distance of $d$ = 115 [nm] (see Fig. 2e) at the central region which is connected to the toroidal rim, similar simplified models have been used previously (1, 2). We consider the state where nanotube and sheet coexist. Using Eqs. (S2) and (S3), we can define the total energy of coexisting nanotube and DMS as:

$$E_{tot}^{(co)} = E_{sh}^{(co)} A_{sh}^{(co)} + E_{nt}^{(co)} A_{nt}^{(co)} \qquad (S24)$$

Where $E_{sh}^{(co)}$ and $E_{nt}^{(co)}$ are the sum of adhesion and bending energy of coexisting DMS and nanotube, respectively. The total area $A$ is defined as the sum of the coexisting DMS and membrane nanotube areas $A = A_{sh}^{(co)} + A_{nt}^{(co)}$. We then build coexistence energy landscape by calculating the difference of the total energy of coexistence $E_{tot}^{(co)}$ with the total nanotube energy $E_{tot}^{nt}$, which reads

$$\Delta E_{co} = E_{tot}^{(co)} - E_{tot}^{nt} \qquad (S25)$$

We re-scaled the coexistence energy difference by sphere bending energy $\Delta \bar{E}_{co} = \Delta E_{int}/8\pi\kappa$. See, Fig. 5a-c for several examples of the coexistence energy landscape.

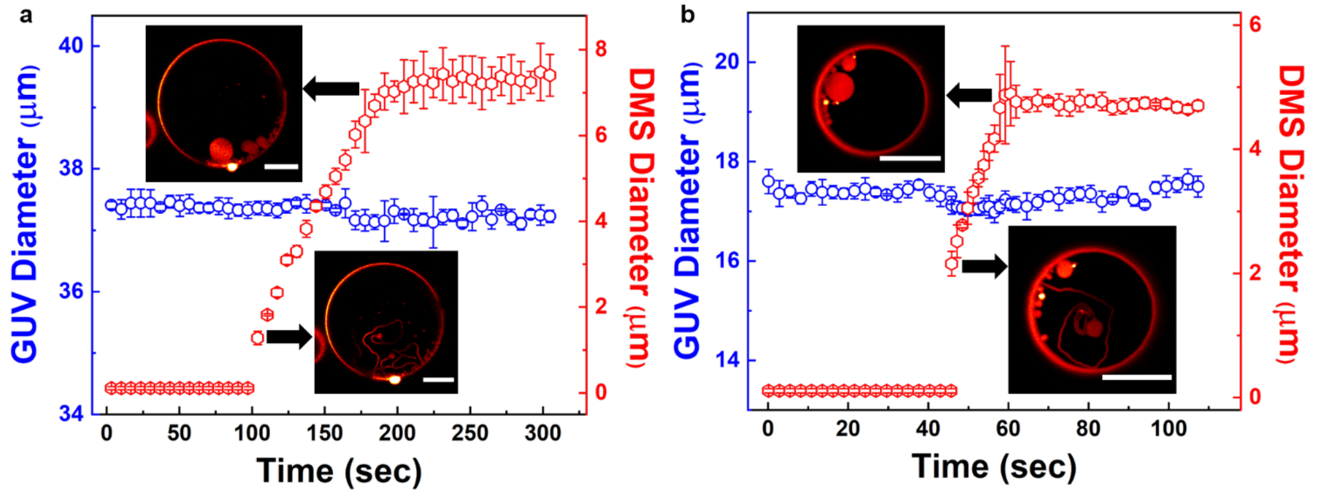


**Figure S6. Two examples showing the time dependence of GUV and DMS diameter change during the nanotube-to-sheet transformation process.** The open blue circles represent the individual GUV diameter and the open red symbols indicate individual DMS diameter during the transformation process captured by confocal time-lapse images. The full transformation process took about 110 seconds for (a) and 15 seconds for (b), as indicated by the time from onset of DMS diameter change to reaching the plateau (see insets). The GUV diameter stayed approximately constant during the entire transformation process suggesting that the transformation occurs at constant area constraint and no exchange of lipid between the tubes/DMS and the GUV. Scale bars: 10 μm.

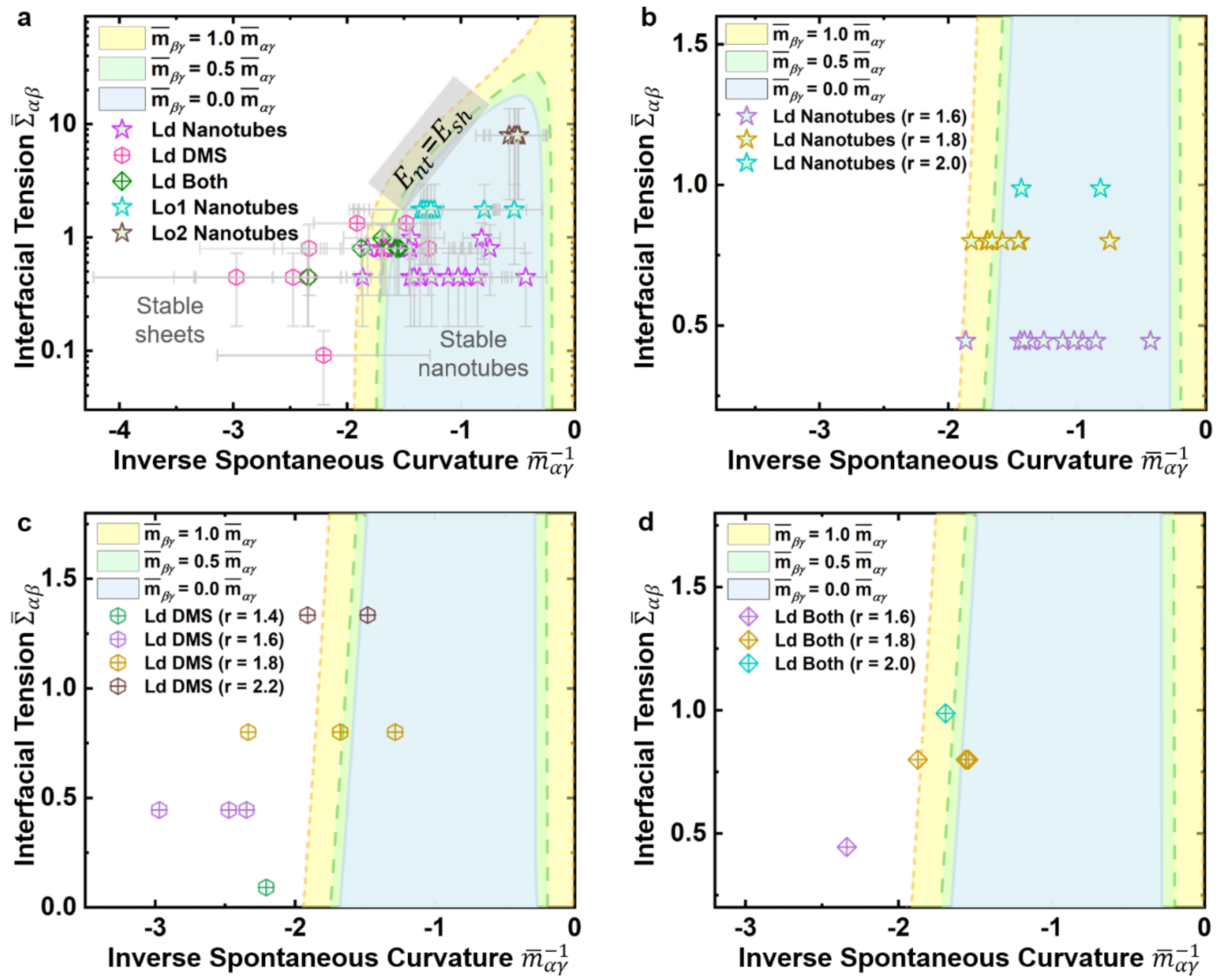

**Figure S7. Full theoretical morphology diagram and experimental data acquired for varied condensate-to-membrane affinity across different deflation ratios (r).** (a) Full morphology diagram with error bars for all the data points are shown in light grey. Three different membrane compositions termed as Ld, Lo1 and Lo2 were investigated, with Ld corresponding to liquid-disordered phase, and Lo1 and Lo2 to liquid-ordered phase exhibiting higher bending rigidities. Increasing the bending rigidity allows exploring the upper range of the morphological diagram. However, distinguishing different contributions (of the bending rigidity, condensate-membrane affinity, spontaneous curvature) is complex. For example, changes in membrane composition could alter not only (i)the bending rigidity, but also (ii) the affinity to the two phases (either in a similar or different way), which can alter (iii) the spontaneous curvature of the two membrane segments. (b-d) Variation of condensate-to-membrane affinity based on different deflation ratios are shown for Ld GUVs exhibiting only nanotubes (b), only DMS (c), and both structures (d). The condensate affinity to the membrane is enhanced by increasing PEG concentration in the PEG-rich droplet through higher deflation (larger r), as a consequence shifting the explored region to higher rescaled interfacial tension.

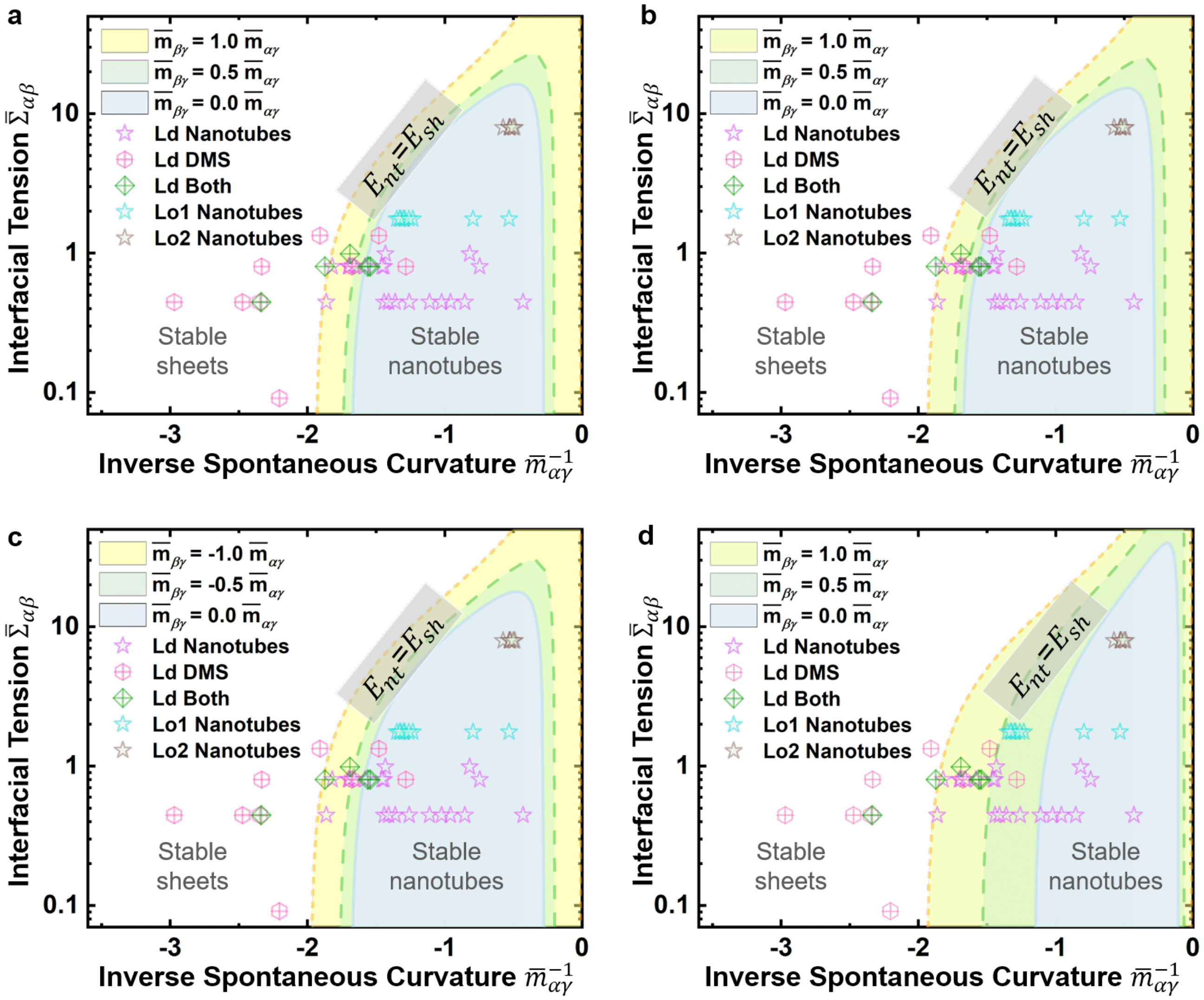


**Figure S8.** The morphology diagram is constructed based on Eq. (S23) and the model parameters presented in Table S1, S2: Intrinsic contact angle $\theta_{in} = 70°$ in (a) and $\theta_{in} = 80°$ in (b), which are close to the upper and lower limits of the experimentally observed intrinsic contact angles, respectively. Morphology diagram for positive spontaneous curvature $m_{\beta\gamma} > 0$ in **(c)**, and for a nonuniform spontaneous curvature $m_{\alpha\gamma} = cm_{\beta\gamma}$ of the nanotubes in (d) with c= 0, 0.5 and 1, see Eq. (S14). The dashed lines show the transition lines where the DMS and membrane nanotubes have the same energy. Each transition line corresponds to a different spontaneous

curvature of the $\beta\gamma$ segment, for which no experimental values are available. Below the transition lines, the nanotubes are stable and above these line the DMSs are stable.

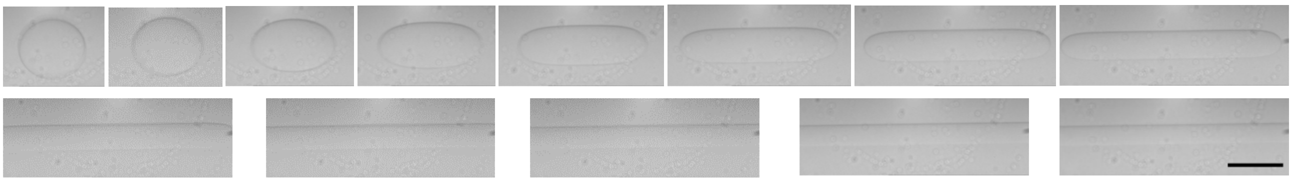

**Figure S9. ATPS interfacial tension measurement.** The images show a PEG-rich droplet inside the bulk dextran-rich phase at rotation speeds of 500, 1000, 2000, 3000, 4000, 5000, 6000, 7000, 8000, 9000, 10000, 11000 and 12000 rpm, with an initial polymer weight fraction of ($w_d$, $w_p$) = (0.0679, 0.0432) prior to phase separation. The long axis of the droplet is parallel to the rotation axis, the intrinsic interfacial tension (listed in Table S3) can be assessed when the length of the droplet exceeds 4 times of its equatorial diameter at high angular frequencies. Scale bar: 0.5 mm.

| Osmolarity Ratio (r) | 1.4 | 1.6 | 1.8 | 2.0 | 2.2 |
|---|---|---|---|---|---|
| Weight Fraction ($w_d$, $w_p$) | 0.0559, 0.0356 | 0.0596, 0.0379 | 0.0641, 0.0409 | 0.0679, 0.0432 | 0.0706, 0.0449 |
| Interfacial Tension ($\Sigma_{pd}$, μN/m) | 0.31 ± 0.02 | 3.13 ± 0.01 | 8.00 ± 0.14 | 11.59 ± 0.30 | 15.66 ± 0.27 |
| Nanotube Daimeter $D_{nt}$ [nm] | 109 ± 35 | 93 ± 37 | 78 ± 30 | 72 ± 10 | |

**Table S3. ATPS interfacial tension at different initial polymer weight fractions.**

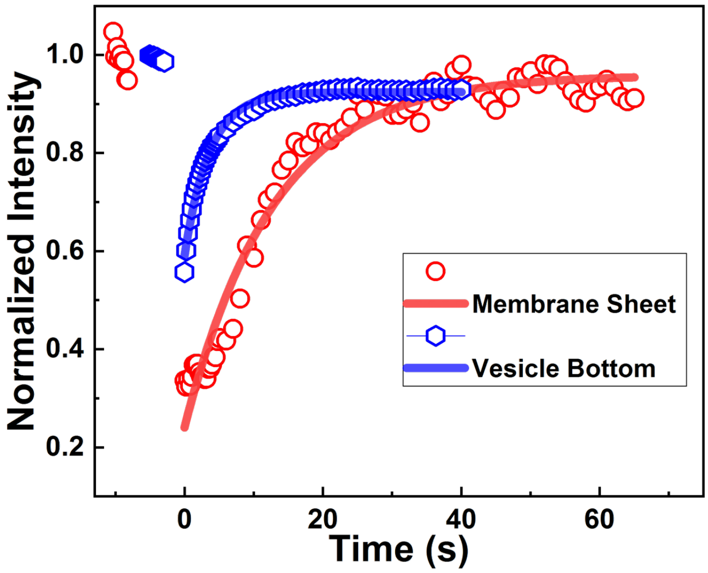


**Figure S10. Fluorescence recovery comparison between a DMS and its corresponding basal membrane of the mother vesicle.** The hollow red circles represent the normalized FRAP data of the DMS, and the red curve represents the corresponding fitted data. The hollow blue hexagons represent the normalized FRAP data of the corresponding mother vesicle membrane, and the blue curve represents the fitted data. Time 0 corresponds to the first frame immediately after photobleaching. The fluctuations in DMS fluorescence intensity at the latter half of the curve are caused by its displacement both in the lateral and axial plane; due to the same reason accurate

statistical fluorescent intensity analysis cannot be performed. Keeping the bleach areas of the same size (bleach spot diameter of 4 μm), the half fluorescence recovery time for the basal mother vesicle membrane is about 3.6 s while that for the DMS is about 12.9 s for this GUV.

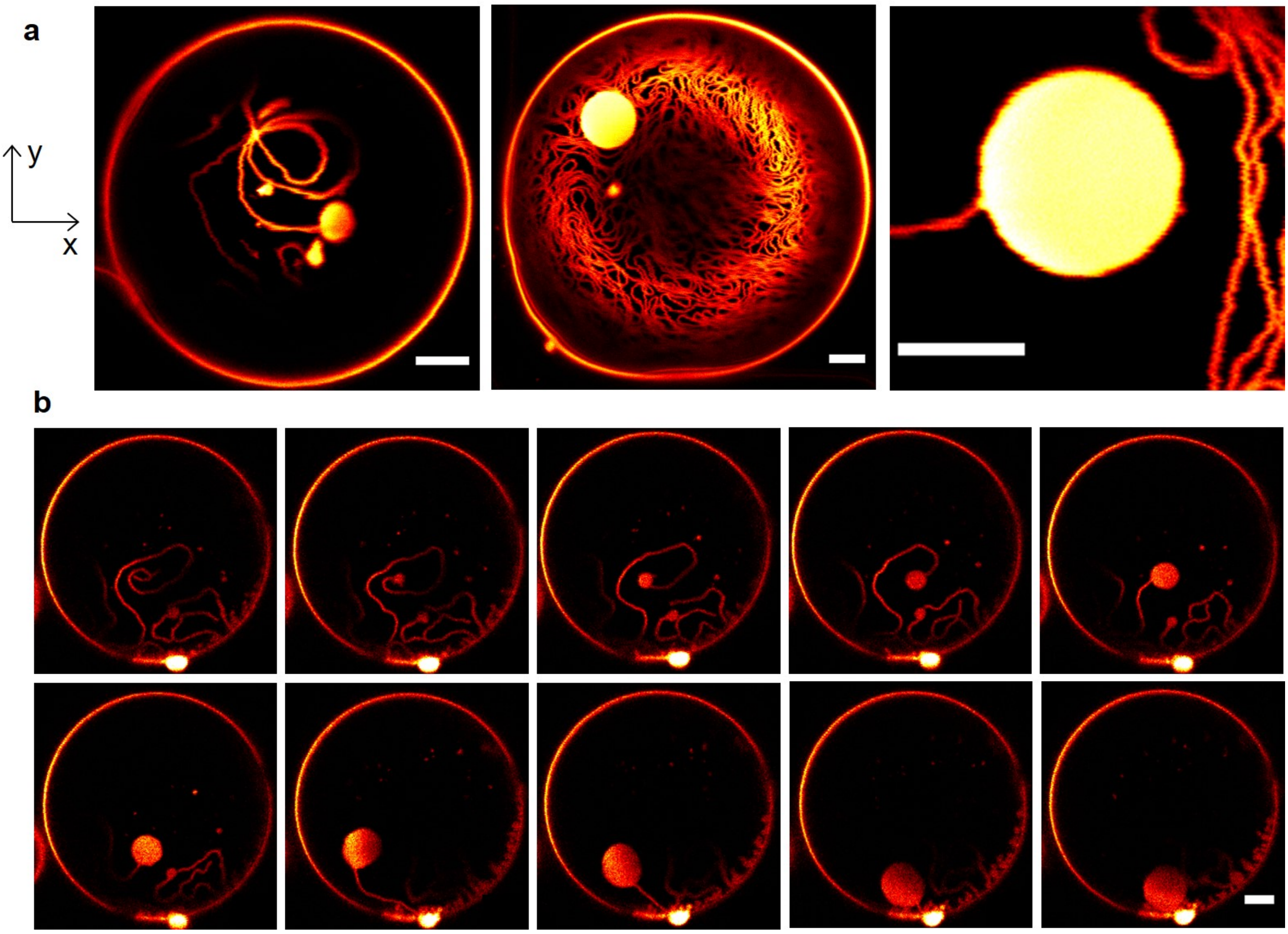


**Figure S11. (a) Examples of DMSs originating from the flattening end of a nanotube.** The DMSs are in general connected to one nanotube, which serves as the sheet area reservoir. **(b)** Complete sequence corresponding to pathway shown in Fig. 4a. Scale bars: 5 μm.

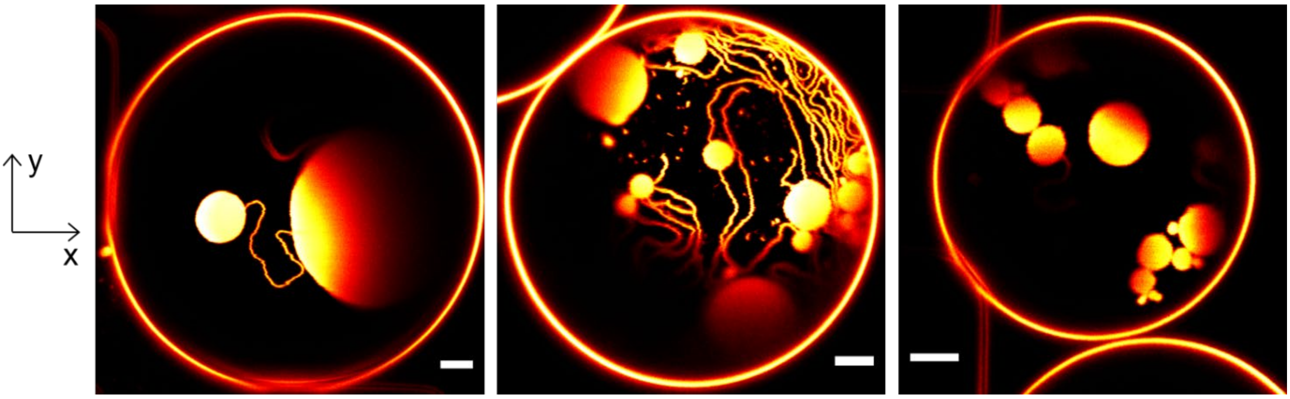


**Figure S12. GUVs with DMSs that have originated from both pathways shown in Fig. 4b, namely from the end of a nanotube and from coalescence of nanotubes at the three-phase contact line (where the ATPS interface meets the mother vesicle membrane).** The two types of DMSs can either be connected with a nanotube or locate at different sites at the interface. Such GUVs were witnessed only very few times in a microfluidic chip containing several hundreds of trapped GUVs. Scale bars: 5 μm.

| GUV Number | Bending Rigidity, $k_BT$ |
|---|---|
| 1 | 11.0 ± 2.8 |
| 2 | 18.7 ± 5.8 |
| 3 | 11.5 ± 3.3 |
| 4 | 16.2 ± 3.6 |
| 5 | 11.8 ± 2.9 |
| 6 | 15.3 ± 2.6 |
| 7 | 16.0 ± 4.8 |
| 8 | 16.6 ± 5.5 |
| 9 | 15.9 ± 5.3 |
| 10 | 15.1 ± 4.8 |
| 11 | 15.9 ± 5.9 |
| Average Value | 14.9 ± 4.3 |

**Table S4. Bending rigidity data obtained from fluctuation analysis.**

**DMS- and knot-free GUVs at high osmolarity ratios.**

A small fraction of the GUVs which did not develop DMSs and entanglement by nanotube knots, exhibited only free nanotubes at the two-phase interface. There are several reasons for this. The tube-to-sheet transformation or nanotube knots formation both require time and are competitive processes. Our observations were limited to 2.5 – 3 hours, but incubations overnight did result in more DMSs, even though we cannot exclude fluctuations in the osmolarity conditions due to sample evaporation over such long periods. Another reason relates to the polymer encapsulation efficiency in GUVs. Even though all GUV internal concentrations are osmotically balanced to the same level after each deflation step, the different GUVs might feature slightly different initial polymer concentration after electroformation (3, 4), see also Fig. S13. The initial GUV excess area, volume and tension are also not well controlled by the preparation protocol. Thus, upon deflation, some GUVs deflate less than others resulting in smaller number of formed nanotubes, i.e. lesser amount of excess area stored in nanotubes, which may be insufficient for tube-to-sheet transformation. We attempted to follow GUVs with a higher initial polymer concentration by incorporating FITC labeled dextran into the ATPS to label dextran-rich phase (Fig. S13). However, because of the relatively quick bleaching of FITC, we were unable to quantitatively compare fluorescence intensity over the hour's long observation experiments.

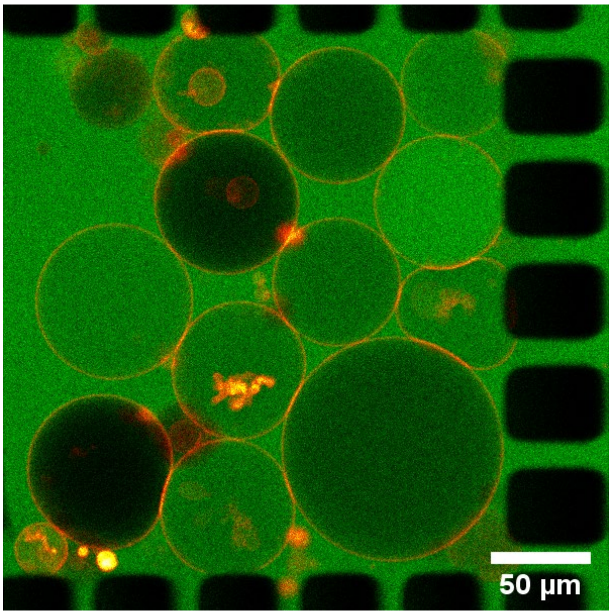


**Figure S13. ATPS GUVs showing different internal FITC fluorescent intensities after preparation.** 0.5 mol% of dextran molecules were labeled with FITC (green) and encapsulated into the GUVs through the same vesicle fabrication protocol, vesicle membrane is labeled with ATTO 647N DOPE (hot red). The variance in internal FITC intensity reflects different amount of ATPS encapsulated, which can result in different amount of excess membrane areas after deflation.

## Supplementary Movies

**Movie S1: DMS at ATPS interface viewed by transmission light microscopy.** ATPS GUVs (with 0.5% ATTO 647N DOPE) were diluted and deflated directly with 5~7 mM sucrose (r is between 1.3 and 1.4) added to the initial isotonic solution and screened for DMS formation. The movie was taken in phase contrast mode with a PCO camera (pco.edge) and a 40× /NA 0.65 objective at a frame rate of 20 fps on an inverted microscope (Zeiss Observer.D1) after vesicle incubation in a hypertonic solution. Manual adjustment of the focus in Z axis was needed in order to display the DMS which appears as a dense black circle (because of the refractive index difference between the two phases and external medium) at the ATPS interface. The PEG-rich and dextran-rich phase each appears as an optical light phase (top) and an optical dense phase (bottom). The movie is played with a frame rate of 50 fps. Scale bar is 5 μm.

**Movie S2: Tube-to-sheet transformation via end region flattening of the nanotube.** The movie displays the whole tube-to-sheet transformation process initiated from end region flattening of the nanotube which took about 11 seconds. Movie S2 corresponds to the GUV in Fig. 4a. The movie is played with a frame rate of 100 fps.

**Movie S3: Tube-to-sheet transformation via nanotube branching.** The movie displays the whole tube-to-sheet transformation process initiated from nanotube branching which took about 110 seconds. Movie S3 corresponds to the GUV in Fig. 4a. The movie is played with a frame rate of 100 fps.

**Movie S4: Membrane sheet-to-nanotube back transformation trajectory.** The movie displays the whole sheet-to-tube transformation process which took about 150 seconds. Movie S4 corresponds to the GUV in Fig. 4a. The movie is played with a frame rate of 100 fps.

**Movie S5, Movie S6: 2D STED xy-t scans of nanotube knots.** Two exemplary nanotube knots movies captured by 2D STED, movie S5 features an aster-like knot and movie S6 features a bundle-like knot, both structures were tightly bounded together and could not disassociate, the bounded nanotubes are unlikely to go through further shape transformations. Movie S5 and S6 correspond to nanotube knots in Fig. 5f and 5g. The movies are played with a frame rate of 15 fps.

**Movie S7: Enlarged nanotube knot video for Figure 5h.** The enlarged STED xy-t scan shows nanotube fluctuates on the interface while keeping the nanotube knot shape intact. The movie is played with a frame rate of 5 fps.

**Movie S8: Confocal and 3D STED xz-t scans of nanotube knots.** A thin layer of nanotubes is accompanied by a large knot in the center of the frame. Fine structures of the nanotube knots cannot be obtained by 3D STED indicating the gap dimension between the nanotubes is below 3D STED axial resolution which is around 110 nm, considering the size of the nanotubes which are around 100 nm, this indicates the nanotubes are tightly squeezed together thus appears like an integrated structure. The center of focus appears to be constantly fluctuating as the structures are mobile, the nanotube size seems to be larger in dimension on the Z axis in confocal movie due to its poor axial resolution. Confocal scans (left) are shown for comparison with STED scans (right). The movies are played with a frame rate of 12 fps.

## SI References:

1. R. L. Knorr, R. Dimova, R. Lipowsky, Curvature of double-membrane organelles generated by changes in membrane size and composition. *PloS one* **7** (2012).
2. F. Campelo *et al.*, Sphingomyelin metabolism controls the shape and function of the Golgi cisternae. *eLife* **6**, e24603 (2017).
3. L. M. Dominak, C. D. Keating, Macromolecular crowding improves polymer encapsulation within giant lipid vesicles. *Langmuir* **24**, 13565-13571 (2008).
4. L. M. Dominak, D. M. Omiatek, E. L. Gundermann, M. L. Heien, C. D. Keating, Polymeric crowding agents improve passive biomacromolecule encapsulation in lipid vesicles. *Langmuir* **26**, 13195-13200 (2010).
